# Effect of different modalities of artificial intelligence rehabilitation techniques on patients with upper limb dysfunction after stroke—A network meta-analysis of randomized controlled trials

**DOI:** 10.3389/fneur.2023.1125172

**Published:** 2023-04-17

**Authors:** Yu Zhu, Chen Wang, Jin Li, Liqing Zeng, Peizhen Zhang

**Affiliations:** ^1^School of Sports Medicine and Rehabilitation, Beijing Sport University, Beijing, China; ^2^Linfen Central Hospital, Linfen, Shanxi, China

**Keywords:** artificial intelligence rehabilitation, stroke, upper limb function, randomized controlled trials, network meta-analysis

## Abstract

**Background:**

This study aimed to observe the effects of six different types of AI rehabilitation techniques (RR, IR, RT, RT + VR, VR and BCI) on upper limb shoulder-elbow and wrist motor function, overall upper limb function (grip, grasp, pinch and gross motor) and daily living ability in subjects with stroke. Direct and indirect comparisons were drawn to conclude which AI rehabilitation techniques were most effective in improving the above functions.

**Methods:**

From establishment to 5 September 2022, we systematically searched PubMed, EMBASE, the Cochrane Library, Web of Science, CNKI, VIP and Wanfang. Only randomized controlled trials (RCTs) that met the inclusion criteria were included. The risk of bias in studies was evaluated using the Cochrane Collaborative Risk of Bias Assessment Tool. A cumulative ranking analysis by SUCRA was performed to compare the effectiveness of different AI rehabilitation techniques for patients with stroke and upper limb dysfunction.

**Results:**

We included 101 publications involving 4,702 subjects. According to the results of the SUCRA curves, RT + VR (SUCRA = 84.8%, 74.1%, 99.6%) was most effective in improving FMA-UE-Distal, FMA-UE-Proximal and ARAT function for subjects with upper limb dysfunction and stroke, respectively. IR (SUCRA = 70.5%) ranked highest in improving FMA-UE-Total with upper limb motor function amongst subjects with stroke. The BCI (SUCRA = 73.6%) also had the most significant advantage in improving their MBI daily living ability.

**Conclusions:**

The network meta-analysis (NMA) results and SUCRA rankings suggest RT + VR appears to have a greater advantage compared with other interventions in improving upper limb motor function amongst subjects with stroke in FMA-UE-Proximal and FMA-UE-Distal and ARAT. Similarly, IR had shown the most significant advantage over other interventions in improving the FMA-UE-Total upper limb motor function score of subjects with stroke. The BCI also had the most significant advantage in improving their MBI daily living ability. Future studies should consider and report on key patient characteristics, such as stroke severity, degree of upper limb impairment, and treatment intensity/frequency and duration.

**Systematic review registration:**

www.crd.york.ac.uk/prospero/#recordDetail, identifier: CRD42022337776.

## Introduction

Globally, stroke is the leading cause of disability in adults, often resulting in symptoms such as muscle weakness, sensory deficits, spasticity, balance problems, reduced dexterity, communication difficulties and cognitive impairment (1). Evidence shows that 40% of people with a stroke still have upper limb impairment, which can lead to limited movement (2–4). Meanwhile, only 5 to 20% of stroke survivors recover full upper limb function, 25% recover partial upper limb function, and 60% have a complete loss of upper limb function (5). Consequently, reduced motor function of the upper limb (e.g., reaching and grasping) can have a significant negative impact on the ability to perform activities of daily living (ADLs) (e.g., eating, dressing and washing) (6).

One study found changes in the affected upper limbs were usually more apparent than in the affected lower limbs (7), including functional limitations in the affected arms and slow, uncoordinated movements of the hands (8, 9). Another study found that subjects with strokes had difficulty performing reaching tasks and movement when manipulating objects due to changes in timing and coordination as well as abnormal postural adjustments (10, 11) or were unable to control grip and fingertip strength (12, 13). Due to a combination of physical, cognitive and perceptual problems, those who have suffered strokes often have difficulty participating in family, work and community life and performing ADLs such as feeding, dressing and grooming (14).

Functional performance of the affected upper limbs can be improved if the subject with stroke has adequate opportunities for exercise. Different techniques and methods can be used in rehabilitation management (e.g., physiotherapy, occupational therapy, conductive education, splinting, pharmacotherapy and surgery) and specific techniques (e.g., neurodevelopmental therapy (NDT) or constraint-induced movement therapy (CIMT) (15–19). However, no strong evidence exists about successful treatment using any of these techniques or methods.

With the rapid development of rehabilitation management technology, artificial intelligence (AI) technology, represented by rehabilitation robots (RT), has received widespread attention from medical researchers (20). AI is defined as the study of disciplines that enable computers to simulate human thought processes and intelligent behaviors (such as learning, reasoning, thinking, planning, etc.). This study mainly includes assessing the principles by which computers are manufactured to replicate and realize human brain intelligence and can achieve higher-level applications (21). Meanwhile, the upper limb RT is a medical robot that facilitates the recovery of upper limb function by driving the patient through repetitive upper limb movement training with mechanical assistance (22). Additionally, brain-computer interface (BCI) electrical stimulation training is a new method of central neurological intervention that collects signals from the patient's brain during motor imagery tasks, converts them into computer commands, and applies electrical stimulation to the paralyzed limb. This enables the establishment of a “central-peripheral-central” closed-loop rehabilitation training model that promotes central re-modeling and peripheral control, thereby facilitating the recovery of motor function (23, 24).

Remote rehabilitation (RR) is a rehabilitation model that uses Internet communication technology to achieve inter-temporal treatment between medical workers and patients, which is convenient, fast and without time and space boundaries, and supports the continued rehabilitation training of patients after discharge from hospital (25). Intelligent rehabilitation (IR) is a new type of intelligent biofeedback therapy device which uses two-dimensional virtual games as biofeedback to conduct interactive training with patients through visual, auditory and tactile forms. IR can be used to assess and train patients' manual motor and sensory functions and to rehabilitate people with cognitive impairment (26). Similarly, virtual reality (VR) technology is an effective tool for stroke rehabilitation, using computers to generate a virtual environment that simulates reality and uses a variety of sensing devices to “immerse” the user in that environment, enabling the user to interact naturally with the virtual environment (27). Combining the characteristics of the AI technologies mentioned above reveals that BCI, RR and VR share common ground regarding training characteristics. Moreover, IR contains the virtual interactive scenarios found in VR technology. However, the training principles of RR, RT and BCI are different. RT emphasizes mechanical assistance for hemiplegic upper limbs, BCI emphasizes central neural integration, based on central integration and peripheral control to assist rehabilitation training, and RR emphasizes online 5G technology to provide online rehabilitation guidance for home rehabilitation patients. Non-invasive VR, on the other hand, detects the thought activity of the brain through a non-implantable device, and the signal is substantially attenuated as it passes through the skull, resulting in low signal intensity and accuracy. Invasive BCIs require the implantation of electrodes into the cerebral cortex to enable interaction and thus have sufficiently precise and risky properties.

Researchers Erosy and Iyigun demonstrated that virtual and real boxing training significantly restored motor function in the hemiplegic upper limbs of subjects with stroke, which supports the effectiveness of virtual boxing training (28). Rodríguez-Hernández et al. showed that VR was more effective than traditional rehabilitation methods in improving stroke patients' quality of life (29). Also, another study found that combining traditional physical fitness with VR technology increased patients' interest in rehabilitation and made them more engaged, leading to better clinical outcomes (30). Previous research confirms that BCI combined with other treatments such as robotic orthoses, mobile robots, VR devices and functional electrical stimulation (FES) effectively improve limb function in subjects with stroke (31). Another study confirmed that RR technology, based on large data platforms, can enable patients to access quality rehabilitation medical services at home, provide long-term rehabilitation support for patients and their families, reduce the gap between in- and out-patients and facilitate function recovery as well as the continuity of rehabilitation treatment (32).

However, the aforementioned studies all investigated the effect of a single AI technique on upper limb function amongst subjects with strokes. Network meta analysis (NMA) may provide a way to address this issue. In randomized controlled trials (RCTs), a quantitative summary of the “network of evidence” is achieved by combining the direct and indirect effects of three or more interventions compared with the same comparative intervention (usually a control or no-treatment intervention) (33). This is also referred to as a multiple treatment comparison (34). In this way, NMA can quantitatively combine evidence on the effectiveness of interventions directly compared in the same RCTs (direct comparison) and interventions from different RCTs with a common comparator (indirect comparison) (33).

At present, most of the meta-analyses at home and abroad investigating AI rehabilitation focus on the single RR and the effects of BCI, VR and RT on the upper limb function and motor function of subjects with stroke. There are few reports on NMA analysis of various AI techniques used in rehabilitating the upper limb function of subjects with stroke (35–38). There is only one NMA analysis on the use of upper limb RT in upper limb motor function in subjects with stroke. The study observed its effect on upper limb function in subjects with stroke by drawing multiple comparisons among different models of upper limb RT. Its indirect comparison showed that none of the types of upper limb RT were better or worse than any other RT, nor did it provide clear evidence to support the choice of a specific type of robotic device to facilitate arm recovery (39).

The present NMA analysis integrates all new AI technologies based on previous studies. Therefore, this study aimed to provide a systematic overview of current RCTs of different modalities of AI techniques and assess their relative effectiveness using an NMA. We aimed to assess the relative influence of different modalities of AI techniques on ADLs, hand/arm function and overall upper limb motor function amongst subjects with strokes and explore the safety of these techniques.

## Methods

### Study enrollment and reporting

The protocol was based on the preferred reporting items for systematic reviews and meta-analyses protocols (PRISMA guidelines 2020) (40). PRISMA extension statements were used to ensure that all aspects of methods and results were reported (41). The protocol is registered in PROSPERO [registration number CRD42022337776 (https://www.Crd.york.ac.uk/prospero/#recordDetails)].

### Search strategy

The study was conducted using PubMed, Embase, Web of Science, the Cochrane Library and CNKI, Wanfang and VIP databases in English and Chinese. A comprehensive and reproducible literature search was undertaken up to September 2022. We developed a search strategy for a combination of thematic terms and free terminology based on the Population, Intervention, Comparison, Outcome, Study Design (PICOS) principles. The specific search protocol included various medical topics and free-text terms related to stroke, cerebrovascular disease, upper limb and hand dysfunction and AI to obtain a broad range of literature for further analysis. PubMed is used as an example, and the specific search strategy is provided in Supplementary Table S1.

### Inclusion criteria

The inclusion criteria were as follows: (1) Population: the diagnostic criteria for stroke were met by the Classification of Cerebrovascular Diseases, and a diagnosis of cerebral infarction or cerebral hemorrhage was made by cranial CT or MRI (2, 42, 43). Intervention: six AI technologies (RT, BCI, RR, IR, VR, RT + VR) were used as intervention methods, alone or in combination with artificial intelligence rehabilitation; (3) Comparison: the control group received only conventional rehabilitation or any of the above intervention groups; (4) Outcome: primary outcome: FMA-UE-Total, secondary outcome: FMA-UE-Distal, FMA-UE-Proximal, ARAT and MBI; (5) Study design: only RCTs were included in this study.

### Exclusion criteria

The exclusion criteria were as follows: (1) other neurological disorders; (2) no accurate diagnosis or inconsistent with the included diagnosis; (3) no outcome indicators or inconsistent with the study indicators; 4) interventions inconsistent with the inclusion criteria; (5) duplicate published studies or incomplete study data even after contacting the authors; and (6) systematic reviews, meta-analyses, theoretical studies, expert reviews, animal experiments, conference reports, economic analyses or case reports.

### Study selection

EndNote (version X20, Clarivate, Philadelphia, Pennsylvania, USA) was used to process the search records. Two reviewers (JL and CW) independently screened the titles and abstracts against the developed inclusion and exclusion criteria. This was followed by reading the full text to exclude documents that did not meet the inclusion criteria. Finally, the two authors identified the remaining literature for inclusion. During this process, any discrepancies were discussed and resolved by the third author (YZ).

### Data extraction and quality assessment

We completed the data extraction using Microsoft Excel. The data extraction was strictly based on author(s), year of publication, specific information about treatment and control groups and primary and secondary outcome indicators. Any disagreements between the two reviewers (LZ and YZ) were judged by a third reviewer (P.Z. Zhang). Two reviewers (YZ and CW) assessed the potential risk of bias in each study by independently using the Cochrane Risk of Bias Tool (44). We assessed seven areas: random sequence generation, allocation concealment, blinding of participants and personnel, blinding of outcome assessment, incomplete outcome data, selective outcome reporting and other possible biases. Each item was rated as unknown, low, or high risk of bias. The assessment was performed in Review Manager (version 5.3). The reviewer discrepancies were also addressed through discussions with a third reviewer (PZ) (45).

### Statistical analysis

When trials used the same testing procedure (e.g., Barthel Index), we calculated the mean difference (MD) and the corresponding 95% confidence interval (CI). We calculated the standardized mean difference (SMD) using the 95% CI if various outcome measures were used for a given endpoint. For dichotomous endpoints, we determined the index of risk difference (RD) using the 95% CI.

We generated visual forest plots for all direct and indirect comparisons and compiled a relative ranking of each intervention based on the surface under the cumulative ranking line (SUCRA) (46). The SUCRA value calculates the percentage efficacy of each individual intervention compared with the “ideal” treatment. We performed all statistical analyses using the software STATA MP version 16.0 (47).

The network meta-analysis was based on a frequency approach, with weighted least squares for multiple regression with random effects. The method allows full consideration of multi-arm studies and includes restricted maximum likelihood estimates (48).

To test the possible assumptions of the transitivity hypothesis, we assessed global inconsistency utilizing consistency and inconsistency models (48, 49). Transitivity implies no systematic differences between the individual arms of the studies. At the local level, we used a node-splitting approach (48, 50). In addition to quantitative testing, we also qualitatively validated test descriptions that contained significant effect modifiers.

In the network diagram, we assessed the risk of bias between trials for each dimension as a study-level covariate along three dimensions (randomized sequences, hidden and blinded for randomized sequences).

## Results

### Results of study identification and selection

**Figure 1:**
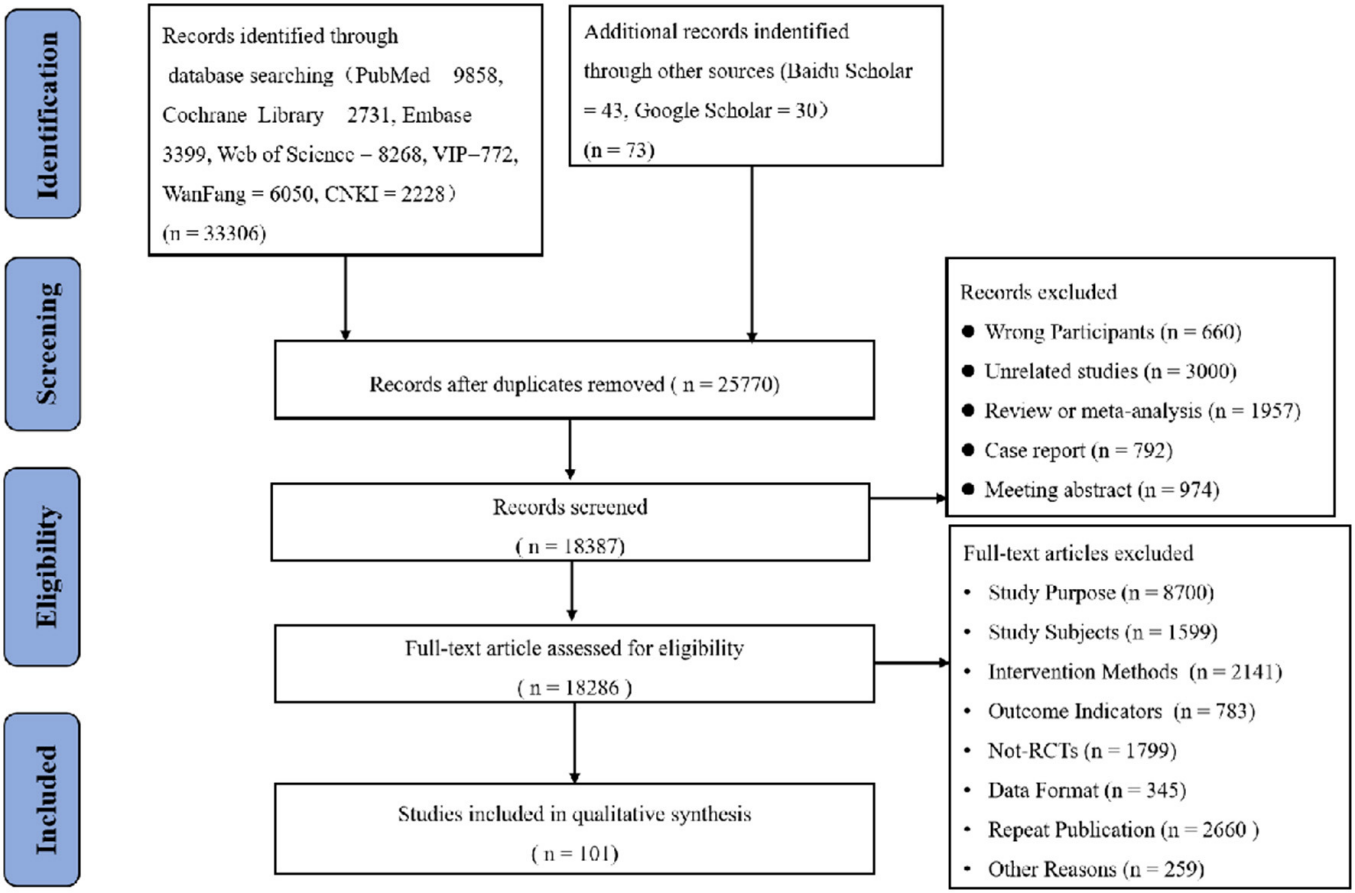
Flow diagram of eligible studies selection process. CNKI, China national knowledge infrastructure; WanFang knowledge servise platform; VIP, Chinese sceintific journals database; n, number of publications.

We included a total of 33,306 studies using the original search terms and 73 original studies in combination with the manual search. After screening for duplicates by de-duplication, we obtained 25,770 original papers. Following this, we obtained 18,387 original papers by applying the inclusion and exclusion criteria combined with abstract reading. After reading the full texts, we obtained 91 original papers (including 8,700 deleted for study purposes, 1,599 deleted for study subjects, 2,141 deleted for intervention methods, 783 deleted for outcome indicators, 1,799 deleted for non-RCTs, 345 deleted for data format discrepancies, 2,660 deleted for duplicate publications and 259 deleted for other reasons). Finally, we included 101 studies (Among them, 91 were filtered and 10 were manually searched according to the purpose of the study) in the risk assessment and NMA. The process of selection of the eligible studies was shown in Figure 1.

### Characteristics of the included studies

We eventually included 101 RCTs, with 2,390 participants in the experimental group (EG) and 2,312 in the control group (CG). Of the 101 studies included, 44 were published in Chinese and 57 in English. The publication period was from 2008 to 2022. The primary outcome indicator was FMA-UE-Total, and the secondary indicators were FMA-UE-Distal, FMA-UE-Proximal, Modified Barthel Index (MBI) and ARAT. Table 1 details the essential characteristics of the included studies.

**Table 1:** Description of the basic characteristics of the included studies.

**Study**	**Location**	**Duration of intervention**	**Type**	**Group**	***n***	**Sex (M/F)**	**Age (years)**	**Ending indicators**
Su et al. (51)	China	4 weeks	Sub-acute stroke	EG:RT+CT	60	14/16	65.53 ± 5.46	FMA-UE, FMA-D, FMA-P, MBI
				CG:CT		15/15	64.97 ± 4.88	
Taravati et al. (52)	Turkey	12 weeks	Chronic stroke	EG:RT+CT	37	14/3	50.94 ± 17.20	FMA-UE
				CG:CT		14/6	55.75 ± 11.61	
Sale et al. (53)	Italy	6 weeks	Sub-acute stroke	EG:RT	53	11/15	67.7(65.8–77.0)	FMA-UE
				CG:CT		11/16	67.7(69.0-78.0)	
He et al. (54)	China	4 weeks	Stroke	EG1:RT+CT	60	26/4	57.67 ± 12.98	FMA-UE, MBI
				CG:CT		26/4	57.53 ± 14.61	
Burgar et al. (55)	USA	6 months	Stroke	EG1:RT-Lo	54	9/10	62.5 ± 2.0	FMA -UE,
				EG2:RT-Hi		9/8	58.6 ± 2.3	
				CG:CT		5/13	68.1 ± 3.3	
de Araújo et al. (56)	Brazil	8 weeks	Chronic stroke	EG:RT	12	5/1	42.83 ± 14.04	FMA-UE, FMA-D, FMA-P
				CG:CT		5/1	52.67 ± 17.84	
Housman et al. (57)	USA	6 months	Chronic stroke	EG:RT	28	7/7	54.2 ± 11.9	FMA-UE, MBI
				CG:CT		11/3	56.4 ± 12.8	
Hsieh et al. (58)	Taiwan	4 weeks	Chronic stroke	EG1:HI-RT	18	4/2	56.04 ± 13.07	FMA -UE,
				EG2:LI-RT		4/2	52.45 ± 1.98	
				CG:CT		5/1	54.00 ± 8.05	
Page et al. (59)	USA	8 weeks	Stroke	EG:RT	16	3/5	59.0 ± 12.9	FMA-UE, MBI
				CG:CT		8/0	58.5 ± 9.5	
Timmermans et al. (60)	Netherlands	6 months	Chronic stroke	EG:RT	22	8/3	61.8 ± 6.8	FMA-UE, ARAT
				CG:CT		8/3	56.8 ± 6.4	
Budhota et al. (61)	Singapore	6 weeks	Sub-acute stroke	EG:RT	44	11/11	56.32 ± 10.37	FMA-UE, ARAT
				CG:CT		14/8	54.59 ± 10.92	
Zhang et al. (62)	China	4 weeks	Stroke	EG:RT+CT	40	14/6	67.3 ± 6.0	FMA-UE, MBI,
				CG:CT+OT		12/8	66.4 ± 4.4	
Sun et al. (63)	China	4 weeks	Stroke	EG:RT+CT	70	21/17	59.11 ± 9.99	FMA-UE, MBI
				CG:CT		17/15	58.06 ± 10.70	
Tomić et al. (64)	Serbia	3 weeks	Stroke	EG:RT	26	12/1	56.5 ± 7.4	FMA-UE, MBI
				CG:CT		9/4	58.3 ± 5.2	
Conroy et al. (65)	USA	12 weeks	Chronic stroke	EG:RT+CT	45	15/8	56.4 ± 12.7	FMA-UE, FMA-D, FMA-P
				CG:CT+TTT		14/8	55.7 ± 10.2	
Fan et al. (66)	China	12 weeks	Acute-stroke	EG:RT+CT	100	29/21	64.46 ± 8.81	FMA-UE, FMA-D, FMA-P, MBI
				CG:CT		30/20	68.00 ± 8.81	
Lee et al. (67)	Korea	2 weeks	Stroke	EG:RT	44	15/7	50.27 ± 11.11	MBI
				CG:CT		14/8	52.32 ± 8.66	
Villafañe et al. (68)	Italy	3 weeks	Stroke	EG:RT+CT	32	11/5	NA	MBI
				CG:CT		10/6	NA	
Zhang et al. (69)	China	4 weeks	Stroke	EG:RT+CT	12	4/2	35.5 ± 9.0	FMA-UE
				CG:CT		5/1	47.0 ± 10.0	
Zhang et al. (70)	China	1 month	Stroke	EG:RT+CT	40	12/8	53.2 ± 9.1	FMA-UE, MBI
				CG:CT		11/9	52.9 ± 8.6	
He et al. (71)	China	12 weeks	Acute-stroke	EG:RT+CT	46	16/7	55.82 ± 11.25	FMA-UE, MBI
				CG:CT+TTT		15/8	54.37 ± 11.02	
Singh et al. (72)	India	4 weeks	Chronic stroke	EG:RT	23	NA	41.1 ± 12.8	FMA-UE, FMA-D, FMA-P, MBI
				CG:CT		NA	42.7 ± 9.3	
Jiang et al. (73)	China	2 weeks	Sub-acute stroke	EG:RT	45	9/14	62.43 ± 11.29	FMA-UE, MBI
				CG:CT		7/15	66 ± 11.51	
Gandolfi et al. (74)	Italy	12 weeks	Chronic stroke	EG:RT	32	12/4	59.31 ± 14.40	FMA -UE
				CG:CT		10/6	59.13 ± 14.97	
Dehem et al. (75)	Belgium	6 months	Stroke	EG:RT	45	11/12	67.1 ± 11.1	FMA-UE,
				CG:CT		10/12	68.6 ± 19.1	
Carpinella et al. (76)	Italy	3 months	Sub-acute stroke	EG:RT	224	63/48	69.5 ± 10.9	FMA-UE, MBI
				CG:CT		64/49	68.5 ± 11.5	
Xu et al. (77)	China	6 weeks	Sub-acute stroke	EG:RT	40	15/5	62.2 ± 10.1	FMA-UE, MBI
				CG:OT		14/6	60.7 ± 10.6	
Huang et al. (78)	Hong Kong	5 weeks	Chronic stroke	EG:Clinic-RT	32	8/8	53.50 ± 13.08	FMA-UE, ARAT, FMA-D, FMA-P
				CG:Lab-RT		12/4	53.06 ± 10.27	
Susanto et al. (79)	Hong Kong	6 months	Chronic stroke	EG:RT	19	7/2	50.7 ± 9.0	ARAT, FMA-UE, FMA-D, FMA-P
				CG:CT		7/3	55.1 ± 10.6	
Carpinella et al. (76)	Italy	3 months	Chronic stroke	EG:RT	38	9/10	67.0 (58.0–70.0)	FMA-UE, FMA-D, FMA-P
				CG:CT		9/10	59.0 (46.0–69.0)	
Dehem et al. (75)	Korea	4 weeks	Chronic stroke	EG1:EXO-RT	38	15/4	49.47 ± 10.88	FMA-UE, MBI
				CG:EE-RT		11/8	54.00 ± 10.01	
Wu et al. (80)	Taiwan	4 weeks	Chronic stroke	EG1:RBAT	42	10/4	55.13 ± 12.72	FMA-UE, FMA-D, FMA-P, MBI
				EG2:TBAT		12/2	57.04 ± 8.78	
				CG:CT		10/4	51.30 ± 6.23	
Abd El-Kafy et al. (81)	Saudi Arabia	12 weeks	stroke	EG:VRT+RT+CT	36	NA	NA	ARAT
				CG:CT				
Hu et al. (82)	China	4 weeks	stroke	EG1:VRT+CT	65	14/8	56.64 ± 11.37	FMA-UE, MBI, ARAT
				EG2:RT+CT		11/11	59.78 ± 11.13	
				EG3:VRT+RT+CT		14/7	57.89 ± 11.88	
Chen et al. (83)	China	2 weeks	stroke	EG:VRT+RT+CT	30	12/3	59.40 ± 11.06	FMA-UE, MBI
				CG:CT		7/8	63.60 ± 10.04	
Wang et al. (84)	China	8 weeks	Chronic stroke	EG1 :RT+CT	48	13/11	56.16 ± 4.52	FMA-UE, MBI
				EG2:VRT+CT		14/10	55.72 ± 4.66	
Gueye et al. (85)	Czech Republic	3 weeks	Sub-acute stroke	EG :VRT	50	14/11	66.56 ± 12.26	FMA-UE
				CG:CT		15/10	68.12 ± 11.97	
Zhao et al. (86)	China	4 weeks	Sub-acute stroke	EG :BCIT	28	13/1	50.1 ± 11.1	FMA-UE, MBI
				CG:CT		12/2	56.16 ± 11.5	
Wang et al. (87)	China	4 weeks	stroke	EG:BCIT+CT	40	9/11	69.05 ± 5.79	FMA-UE, MBI, ARAT
				CG:CT		12/8	67.25 ± 4.78	
Lee et al. (88)	Korea	4 weeks	stroke	EG:BCIT+CT	26	4/9	55.15 ± 11.57	FMA-UE, MBI, WMFT
				CG:CT		6/7	58.30 ± 9.19	
Ang et al. (89)	Singapore	6 weeks	stroke	EG1:BCI-Manus	26	9/2	48.5 ± 13.5	FMA-UE
				EG2:Manus		8/7	53.6 ± 9.5	
Xu et al. (90)	China	8 weeks	stroke	EG:BCI+CT	32	15/1	72.42 ± 8.56	FMA-UE, MBI
				CG:CT		15/1	76.81 ± 9.57	
Liang et al. (91)	China	4 weeks	stroke	EG:BCI+CT	30	12/3	57.94 ± 8.84	FMA-UE, MBI
				CG:CT		9/6	50.06 ± 13.46	
Li et al. (92)	China	8 weeks	stroke	EG:BCI+CT	14	5/2	66.29 ± 4.89	FMA-UE, ARAT
				CG:CT		5/2	60.00 ± 6.30	
Xiang et al. (93)	China	6 weeks	stroke	EG:BCI+CT	94	22/25	58.6 ± 2.7	FMA-UE, MBI
				CG:CT		26/21	60.2 ± 1.9	
Ren and Xie (94)	China	4 weeks	stroke	EG:BCI+CT	60	18/12	41.77 ± 8.65	FMA-UE, MBI
				CG:CT		20/10	40.7 ± 8.15	
Chen et al. (95)	China	4 weeks	stroke	EG:BCI	14	7/0	41.6 ± 12.0	FMA-UE
				CG:CT		5/2	52.0 ± 11.1	
Frolov et al. (2019)	American	2 weeks	stroke	EG:BCI+CT	74	34/21	58.0 ± 12.59	FMA-UE, ARAT
				CG:CT		14/5	58.0 ± 11.11	
Kim et al. (96)	American	4 weeks	stroke	EG:BCI+CT	30	6/9	59.07 ± 8.97	FMA-UE, MBI
				CG:CT		6/9	59.93 ± 9.79	
Mihara et al. (97)	American	2 weeks	stroke	EG:BCI+CT	20	8/2	NA	FMA-UE, ARAT
				CG:CT		4/6	NA	
Zhang et al. (62)	China	8 weeks	stroke	EG:BCIT+CT	30	11/4	60.93 ± 6.76	FMA-UE, MBI
				CG:CT		11/4	57.87 ± 8.61	
Wu et al. (98)	China	8 weeks	Chronic stroke	EG:RR+CT	80	19/21	57.45 ± 9.98	FMA-UE, MBI
				CG:CT		27/13	61.45 ± 9.83	
Xue et al. (99)	China	8 weeks	Chronic stroke	EG:RR+CT	60	NA	NA	FMA-UE, MBI
				CG:CT		NA	NA	
Wang et al. (100)	China	4 weeks	Chronic stroke	EG:RR+CT	38	15/4	53.22 ± 10.65	FMA-UE, MBI
				CG:CT		14/5	53.05 ± 14.83	
Wang et al. (101)	China	48 weeks	stroke	EG:RR+CT	60	24/6	58.0 ± 12	FMA-UE, MBI
				CG:CT		22/8	60.00 ± 9	
Gao et al. (102)	China	12 weeks	stroke	EG:RR+CT	40	12/6	53.2 ± 17.1	FMA-UE, MBI
				CG:CT		14/8	52.2 ± 14.1	
Chen et al. (103)	China	24 weeks	stroke	EG:RR+CT	54	18/9	66.52 ± 12.08	FMA-UE, MBI
				CG:CT		15/12	66.15 ± 12.33	
Chen (104)	China	8 weeks	stroke	EG:RR	44	26/6	65.3 ± 13.2	FMA-UE
				CG:CT		14/8	67.1 ± 10.7	
Maeno et al. (105)	China	24 weeks	stroke	EG:RR	100	31/19	66.50 ± 11.45	MBI
				CG:CT		36/14	66.7 ± 11.76	
Lin et al. (106)	China	4 weeks	stroke	EG:RR	24	2/10	74.6 ± 2.3	MBI
				CG:CT		5/7	75.6 ± 3.4	
Chaiyawat et al. (107)	Thailand	24 weeks	stroke	EG:RR	60	14/16	67 ± 7	MBI
				CG:CT		13/17	66 ± 11	
Redzuan et al. (108)	Malaya	12 weeks	stroke	EG:RR	90	21/23	63.7 ± 12	MBI
				CG:CT		31/15	59.40 ± 11	
Piron et al. (109)	Venezia	4 weeks	stroke	EG:RR	36	11/7	66 ± 7.9	FMA-UE
				CG:CT		10/8	64.4 ± 7.9	
Li et al. (110)	China	12 weeks	stroke	EG:RR	101	28/23	65.69 ± 11.32	FMA-UE, MBI
				CG:CT		27/23	65.51 ± 13.02	
Kwon et al. (111)	Korea	4 weeks	stroke	EG:VR	26	9/4	57.14 ± 15.42	FMA - UE, FMA - P, FMA - D, MBI
				CG:CT		5/8	57.92 ± 12.32	
Chen et al. (112)	China	48 weeks	stroke	EG:VR+RT	49	19/4	64.31 ± 6.11	FMA - UE, FMA - P, FMA - D, MBI
				CG:CT		14/12	66.42 ± 5.6	
Jiang (113)	China	2 weeks	stroke	EG:VRT+RT+CT	40	9/11	63.15 ± 11.79	FMA - UE, FMA- P, FMA - D, MBI
				CG:CT		15/5	65.10 ± 9.14	
Wei (114)	China	3 weeks	Stroke	EG:IR	120	37/23	66.3 ± 5.2	FMA - UE
				CG:CT		35/25	65.7 ± 5.4	
Wang (115)	China	12 weeks	Stroke	EG:IR+CT	110	27/28	64.23 ± 5.95	FMA - UE
				CG:CT		31/24	63.08 ± 6.14	
Prange et al. (116)	Netherlands	6 weeks	Sub-acute stroke	EG:IR	68	17/18	60.3 ± 9.7	FMA - UE
				CG:CT		14/19	58 ± 11.4	
Lee et al. (117)	Korea	2 weeks	Stroke	EG:IR+CT	50	14/11	55.76 ± 13.6	MBI
				CG:CT		12/13	57.88 ± 11.12	
McNulty et al. (118)	Australia	2 weeks	Stroke	EG:IR	41	13/8	59.9 ± 13.8	FMA - UE
				CG:CT		18/2	56.1 ± 17	
Norouzi-Gheidari et al. (119)	Canada	4 weeks	Stroke	EG:VR+CT	18	5/4	42.2 ± 9.5	FMA-UE
Lin et al. (120)	China	4 weeks	Chronic stroke	EG:TG	33	12/4	52.63 ± 10.49	FMA - UE, FMA - P, FMA - D
				CG:CT		16/1	57.47 ± 10.29	
Keskin et al. (121)	Turkey	6 weeks	Stroke	EG:VR+CT	24	4/8	63.6 ± 9.2	FMA - UE
				CG:CT		5/7	63.6 ± 7.1	
El-Kafy et al. (122)	Norway	12 weeks	Chronic stroke	EG:VR+CT	40	16/4	54.46 ± 4.27	ARAT
				CG:CT		15/5	53.32 ± 5.13	
				CG:CT		5/4	57.6 ± 10.5	
Choi et al. (123)	Korea	2 weeks	Ischemic stroke	EG:VR+CT	24	7/5	61 ± 15.2	FMA-UE
				CG:CT		6/6	72.1 ± 9.9	
Anwar et al. (124)	Pakistan	6weeks	Stroke	EG:VR	68	20/14	51.56 ± 7.19	FMA-UE
				CG:CT		14/20	51.35 ± 5.78	
Kim et al. (125)	China	4 weeks	Stroke	EG:VR+CT	60	16/14	70.31 ± 3.81	FMA-UE
				CG:CT		17/13	69.83 ± 3.27	
Xiao et al. (126)	China	4 weeks	Sub-acute stroke	EG:VR	35	10/6	56.12 ± 9.01	FMA-UE, MBI
				CG:CT		12/7	53.67 ± 8.03	
Bo et al. (127)	China	4 weeks	Stroke	EG:VR+CT	60	23/7	64.0 ± 7.74	FMA-UE, MBI
				CG:CT		25/5	62.4 ± 9.77	
Kim (128)	China	2 weeks	Stroke	EG:VR	30	10/5	61.4 ± 8.1	FMA-UE
				CG:CT		9/6	58.8 ± 9.5	
Tian et al. (129)	China	4 weeks	Stroke	EG:VR+CT	60	21/9	57.4 ± 11.34	FMA-UE, MBI
				CG:CT		19/11	58.13 ± 12.57	
Lee et al. (130)	Korea	4 weeks	Stroke	EG:VR+CT	10	3/2	65.2 ± 5.0	FMA-UE
				CG:CT		2/3	66.2 ± 3.4	
Kong et al. (131)	Singapore	3 weeks	Stroke	EG1:VR	102	27/5	58.1 ± 9.1	FMA-UE, ARAT
				EG2:CT		25/8	59.0 ± 13.6	
				CG:CC		25/12	55.8 ± 11.5	
Park et al. (132)	Korea	4 weeks	Stroke	EG:VR+CT	25	7/5	53.5 ± 13.0	FMA-UE, FMA-P, FMA-D, MBI
				CG:CT		8/5	51.5 ± 16.7	
Brunner et al. (133)	Norway	4 weeks	Stroke	EG:VR	130	42/20	62 ± 16.5	ARAT
				CG:CT		35/23	62 ± 11.5	
Choi et al. (134)	Korea	4 weeks	Sub-acute stroke	EG:VR	20	5/5	64.30 ± 10.3	FMA-UE
				CG:CT		5/5	64.70 ± 11.3	
Thielbar et al. (135)	USA	6 weeks	Stroke	EG:VR	14	4/3	54 ± 7	ARAT
				CG:CT		5/2	59 ± 6	
Kiper et al. (136)	Italy	4 weeks	Stroke	EG:VR	44	14/9	63.1 ± 9.5	FMA-UE
				CG:CT		15/6	65.5 ± 14.2	
Lee et al. (137)	Korea	4 weeks	Stroke	EG:VR+CT	24	5/7	58.33 ± 10.17	FMA-UE
				CG:CT		6/6	65.42 ± 9.77	
Kwon et al. (111)	Korea	4 weeks	Stroke	EG:VR+CT	26	9/4	57.15 ± 15.42	FMA-UE, MBI
				CG:CT		5/8	57.92 ± 12.32	
Crosbie et al. (138)	UK	3 weeks	Stroke	EG:VR	18	5/4	56.1 ± 14.5	ARAT
				CG:CT		5/4	64.6 ± 7.4	
Yin et al. (139)	Singapore	2 weeks	Stroke	EG:VR+CT	23	6/5	62 ± 16.3	FMA-UE, ARAT
				CG:CT		10/2	56 ± 11.1	
Zhou et al. (140)	China	12 weeks	Stroke	EG:RR+CT	75	21/16	55.00 ± 5.15	ARAT, FMA-UE, MBI
				CG:CT		20/18	55.97 ± 6.17	
Ögün et al. (141)	Türkiye	6 weeks	Ischemic stroke	EG:VR	65	28/5	61.48 ± 10.92	ARAT, FMA-UE, MBI
				CG:CT		23/9	59.75 ± 8.07	
Nijenhuis et al. (142)	Netherlands	6 weeks	Chronic stroke	EG:IR	20	7/3	58 ± 12.59	ARAT
				CG:CT		3/7	62 ± 11.85	
Wolf et al. (143)	USA	8 weeks	Stroke	EG:RR+CT	99	31/17	54.7 ± 12.2	ARAT, FMA-UE, FMA-P, FMA-D
				CG:CT		25/26	59.1 ± 14.1	
Chen et al. (144)	China	2 weeks	Stroke	EG:VR	36	10/8	57.8 ± 8.4	ARAT, FMA-UE
				CG:CT		10/8	58.4 ± 9.3	
Rand et al. (86)	Israel	5 weeks	Chronic stroke	EG:IR	24	9/4	59.1 ± 10.5	ARAT
				CG:CT		6/5	64.9 ± 6.9	
Qian et al. (145)	China	12 weeks	Sub-acute stroke	EG:RR	24	9/5	54.6 ± 11.3	ARAT, FMA-UE, FMA-P, FMA-D
				CG:CT		6/4	64.6 ± 3.43	
Huijgen et al. (146)	Netherlands	4 weeks	Stroke	EG:RR+CT	17	2/9	69 ± 8	ARAT
				CG:CT		4/1	71 ± 7	

ARAT, action research arm test; AST, arm supporting training; BCI, brain-computer interface; CG, control group; CT, conventional rehabilitation; D, distal; RR, remote rehabilitation training; EG, experimental group; EE, end - efector; EXO, exoskeleton; FMA - UE, fugl-meyer assessment upper extremity; HI, higher intensity; IR, intelligent rehabilitation; LI, lower intensity; Lo, low dose; MBI, modified barthel index; mCIMT, modified constraint induced movement therapy; OT, occupational therapy; P, proximal; RBAT, robot-assised bilateral arm training; RT, robot treatment; RR, remote rehabilitation; TBAT, therapist-based arm training; TTT, transition-to-task therapy; VRT, virtual reality training; WMT, wii-based movement therapy.

### Quality assessment of the included studies

**Figure 2:**
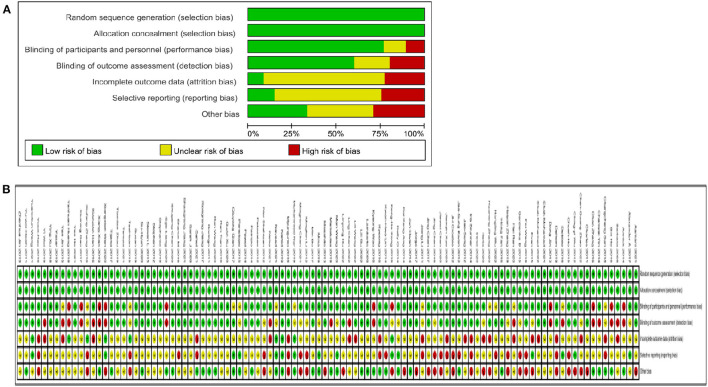
Quality assessment of selected studies by the cochrane risk of bias tool. **(A)** Risl of bias graph: review authors judgments about each risk of bias item presents as percentages across all included studies. **(B)** Risk of bias summary: review authors judgements about each risk of bias item for each included study.

We assessed the risk of bias for each study using the Cochrane Risk of Bias Tool. All studies included RCTs that reported the random sequence generation and allocation concealment with a low risk of bias. Almost two-thirds of the studies were shown to be at low risk in both the blinding of participants and personnel and the blinding of outcome assessment. Of these, only 10% of the studies in the blinding of participants and personnel showed high risk, and 10% showed unclear risk. In the blinding of outcome assessment, ~ 20% of the studies showed high risk, and 20% showed unclear risk. Of the incomplete outcome data, ~ 70% showed unclear risk, 5% showed low risk and 25% showed high risk. In the selective reporting, ~ 15% showed low risk, 60% showed unclear risk and 25% showed high risk. Of the other biases, ~ 35% showed low risk, 30% showed unclear risk and 30% showed high risk. Figure 2 contains detailed information on the risk of bias.

### Results of the network meta-analysis

#### Evidence network diagram

**Figure 3:**
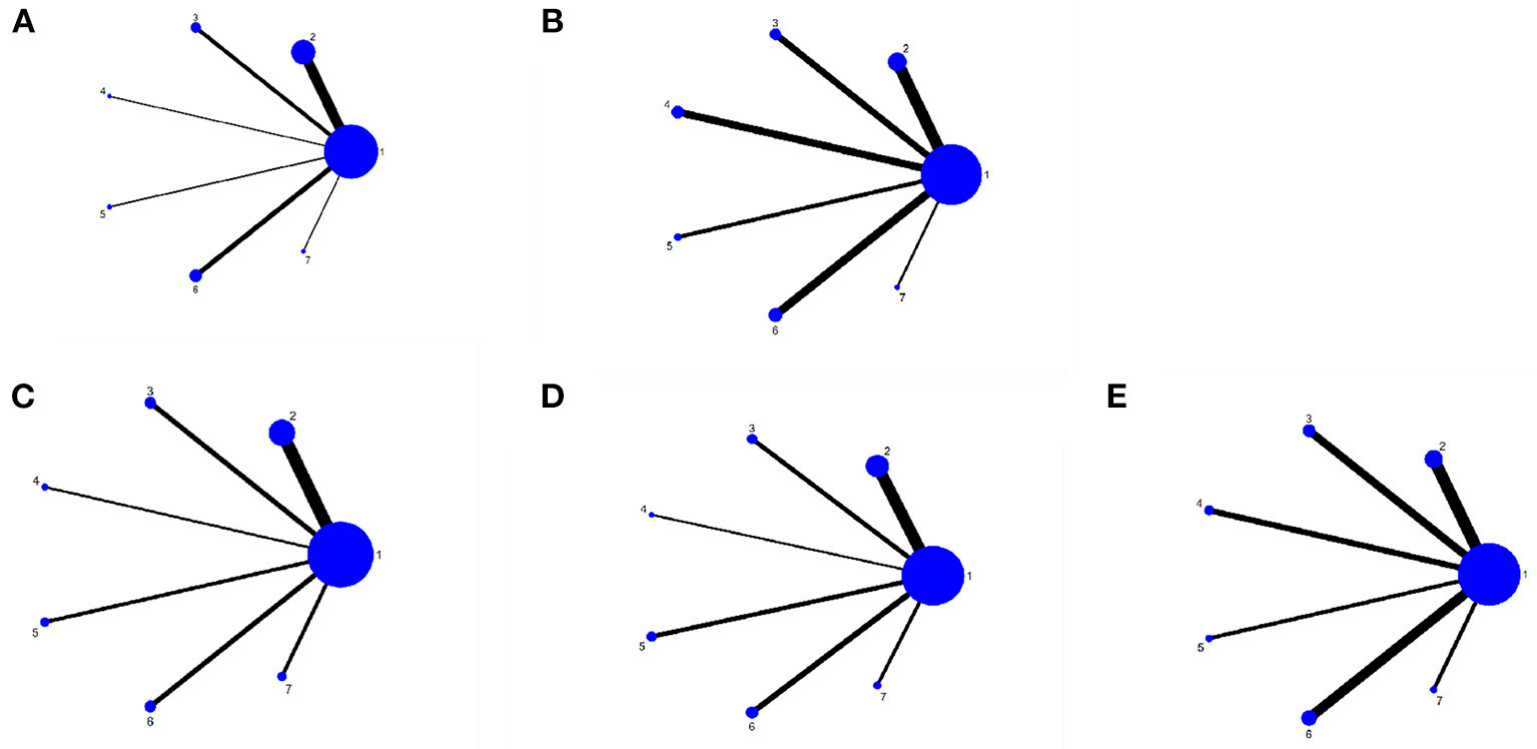
Network meta-analysis diagrams of eligible comparisons. **(A)** FMA-UE-Total, **(B)** MBI, **(C)** FMA-UE-Proximol, **(D)** FMA-UE-Distal, **(E)** FMA-UE-ARAT. Width of the lines is proportional to the number of trial. Size of every circle is proportional to the number of randomly assignes participants (sample size). 1, conventional training (CT); 2, Robot training (RT); 3, Brain-computer interface (BCI); 4, Remote rehabilitation (RR); 5, Intelligent rehabilitation (IR); 6, Virtual reality (VR); 7, Robot training + virtual reality (RT+VR).

A total of 101 studies, 4,702 subjects were included in this study involving six interventions (RT, BCI, RR, IR, VR, RT + VR). A total of 71 studies were included in the network evidence map of the FMA-UE-Total involving the following interventions: CT, RT, BCI, RR, IR, VR, and RT + VR. We included a total of 49 studies in the MBI network evidence map involving interventions such as CT, RT, BCI, RR, IR, VR, and RT + VR. We included 22 studies in the ARAT network evidence map involving CT, RT, BCI, RR, IR, VR, and RT + VR interventions. Moreover, we included a total of 26 studies in the FMA-UE-Proximal network evidence map involving interventions such as CT, RT, BCI, RR, IR, VR, and RT + VR, and we included 28 studies in the network evidence map for the FMA-UE-Distal, involving interventions such as CT, RT, BCI, RR, IR, VR, and RT + VR. Figures 3A–E shows the details of the NMA map.

### Primary outcome

#### FMA-UE-total

We included 71 studies in the FMA-UE-Total in the comparison (51–66, 69–80, 87, 88, 91, 92, 147). As the network evidence map in this study did not form a closed loop, indirect comparisons and inconsistency tests could not be performed (148). However, *P* > 0.05 in the consistency test indicated excellent consistency and stability of the studies.

**Figure 4:**
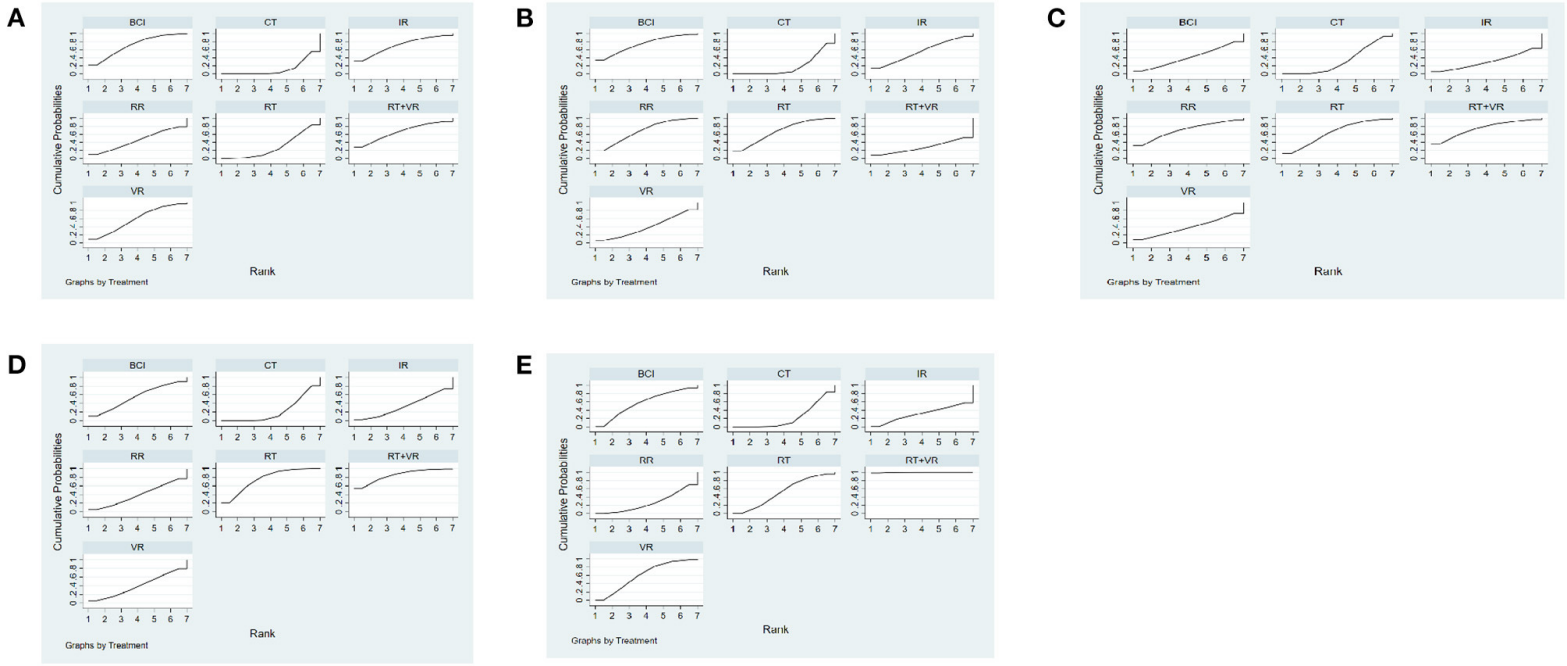
Cumulative probability ranking curve of different interventions. **(A)** FMA-UE-Total, **(B)** MBI, **(C)** FMA-UE-Proximal, **(D)** FMA-UE-Distal, **(E)** FMA-UE-ARAT. The fuller the area under the curve, the more effective it is. 1, conventional training (CT); 2, Robot training (RT); 3, Brain-computer interface (BCI); 4, Remote rehabilitation (RR); 5, Intelligent rehabilitation (IR); 6, Virtual reality (VR); 7, Robot training + virtual reality (RT+VR).

The NMA results showed that all interventions were not statistically significant, indicating that AI rehabilitation techniques did not significantly improve upper limb motor function amongst subjects with strokes (see Table 2). Figure 4A shows the SUCRA rankings for all treatments. Based on the results of the SUCRA analysis, IR [(SMD = 0.02, 95%CI = (−0.40, 0.43)] (SUCRA, 70.5%) was the most effective intervention for improving upper limb motor function amongst subjects with strokes, followed by BCI [(SMD = 0.001, 95%CI = (−0.46, 0.46)] (SUCRA, 69.5%); RT + VR [(SMD = 0.06, 95%CI = (−0.39, 0.51)] (SUCRA, 65.9%); VR [(SMD = 0.06, 95%CI = (−0.36, 0.48)] (SUCRA, 58.1%); RR [(SMD = 0.07, 95%CI = (−0.30, 0.45)] (SUCRA, 45.6%); RT [(SMD = 0.05, 95%CI = (−0.09, 0.20)] (SUCRA, 28.0%) and CT (SUCRA, 12.3%).

**Table 2:** Network analysis results of the FMA – UE -total.

**IR**						
0.02 (−0.40, 0.43)	BCI					
0.02 (−0.50, 0.54)	0.00 (−0.46, 0.46)	RT+VR				
0.08 (−0.33, 0.49)	0.06 (−0.27, 0.39)	0.06 (−0.39, 0.51)	VR			
0.14 (−0.34, 0.62)	0.12 (−0.30, 0.54)	0.12 (−0.40, 0.64)	0.06 (−0.36, 0.48)	RR		
0.21 (−0.16, 0.58)	0.19 (−0.09, 0.47)	0.19 (−0.23, 0.61)	0.13 (−0.14, 0.41)	0.07 (−0.30, 0.45)	RT	
0.26 (−0.07, 0.60)	0.25 (0.01, 0.48)	0.24 (−0.15, 0.63)	0.18 (−0.05, 0.41)	0.13 (−0.22, 0.47)	0.05 (−0.09, 0.20)	CT

### Secondary outcome

#### Modified barthel index

A total of 49 studies were included in the comparison of the MBI. As the network evidence map in this study did not form a closed loop, inconsistency tests could not be performed (148). However, *P* > 0.05 in the consistency test indicated excellent consistency and stability of the studies.

The NMA results showed no significant differences between the interventions for both direct and indirect comparisons, indicating that different modalities of AI technology had no significant effect on improving MBI function in subjects with stroke (see Table 3). Figure 4B shows the SUCRA rankings for all treatments. According to the results of the SUCRA analysis, BCI [(SMD = 0.03, 95%CI = (−0.24, 0.29)] (SUCRA, 73.6%) was the most effective intervention for improving quality of daily life amongst subjects with strokes, followed by RR [(SMD = 0.001, 95%CI = (−0.22, 0.23)] (SUCRA, 68.1%); RT [(SMD = 0.04, 95%CI = (−0.23, 0.31)] (SUCRA, 67.8%); IR [(SMD = 0.05, 95%CI = (−0.25, 0.36)] (SUCRA, 54.6%); VR [(SMD = 0.09, 95%CI = (−0.34, 0.51)] (SUCRA, 41.1%); RT + VR [(SMD = −0.03, 95%CI = (−0.39, 0.34)] (SUCRA, 26.5%) and CT (SUCRA, 18.4%).

**Table 3:** Network analysis results of the MBI.

**BCI**						
0.03 (−0.24, 0.29)	RR					
0.03 (−0.24, 0.29)	0.00 (−0.22, 0.23)	RT				
0.07 (−0.23, 0.37)	0.04 (−0.22, 0.31)	0.04 (−0.23, 0.31)	IR			
0.12 (−0.18, 0.42)	0.10 (−0.17, 0.37)	0.09 (−0.17, 0.36)	0.05 (−0.25, 0.36)	VR		
0.21 (−0.22, 0.63)	0.18 (−0.22, 0.58)	0.18 (−0.22, 0.58)	0.14 (−0.29, 0.56)	0.09 (−0.34, 0.51)	RT+VR	
0.18 (−0.03, 0.39)	0.16 (−0.01, 0.32)	0.15 (−0.01, 0.31)	0.11 (−0.10, 0.32)	0.06 (−0.16, 0.27)	−0.03 (−0.39, 0.34)	CT

#### FMA-UE-proximal

We included 26 studies in the FMA-UE-Proximal comparison. As the network evidence map in this study did not form a closed loop, inconsistency tests could not be performed (148). However, *P* > 0.05 in the consistency test indicates excellent consistency and stability of the studies.

The NMA study showed there were significant differences in direct comparisons between VR + RT [(SMD = 0.43, 95%CI = (0.01, 0.85)], RT [(SMD = 0.32, 95%CI = (0.07, 0.59)] and CT. There were no significant differences in direct and indirect comparisons between the other interventions. This suggests VR + RT and RT effectively improve motor function of the upper limb shoulder and elbow joints amongst subjects with strokes (see Table 4). Figure 4C shows the SUCRA rankings for all treatments. Based on the results of the SUCRA analysis, RT + VR (SUCRA, 84.8%) was the most effective intervention for improving shoulder and elbow joint motor function amongst subjects with strokes, followed by RT (SUCRA, 75.5%), BCI [(SMD = 0.10, 95%CI = (−0.49, 0.69)] (SUCRA, 54.7%); VR [(SMD = 0.01, 95%CI = (−0.62, 0.63)] (SUCRA, 39.6%); RR [(SMD = 0.04, 95%CI = (−0.57, 0.65)] (SUCRA, 38.9%), IR [(SMD = 0.05, 95%CI = (−0.36, 0.45)] (SUCRA, 34.2%) and CT (SUCRA, 22.3%).

**Table 4:** Network analysis results of the FMA - UE - Proximal.

**RT+VR**						
0.11 (−0.38, 0.60)	RT					
0.24 (−0.34, 0.82)	0.13 (−0.34, 0.60)	BCI				
0.34 (−0.26, 0.94)	0.23 (−0.27, 0.72)	0.10 (−0.49, 0.69)	VR			
0.34 (−0.28, 0.96)	0.23 (−0.29, 0.76)	0.10 (−0.51, 0.71)	0.01 (−0.62, 0.63)	RR		
0.38 (−0.20, 0.97)	0.27 (−0.20, 0.75)	0.14 (−0.43, 0.71)	0.05 (−0.54, 0.64)	0.04 (−0.57, 0.65)	IR	
**0.43 (0.01, 0.85)**	**0.32 (0.07, 0.57)**	0.19 (−0.21, 0.59)	0.09 (−0.34, 0.52)	0.09 (−0.37, 0.55)	0.05 (−0.36, 0.45)	CT

RT+VR, RT and CT for direct comparison, showing that the differences are statistically significant.

#### FMA-UE-Distal

We included 28 studies in the FMA-UE-Distal comparison. As the network evidence map in this study did not form a closed loop, inconsistency tests could not be performed. However, *P* > 0.05 in the consistency test indicates excellent consistency and stability of the studies.

The NMA results showed no significant differences between the interventions, compared directly and indirectly, suggesting different modalities of AI techniques did not significantly influence the improvement of wrist joint motor function in the upper limbs of subjects with stroke (see Table 5). Figure 4D shows the SUCRA rankings for all treatments. Based on the results of the SUCRA analysis, RT + VR [(SMD = 0.03, 95%CI = (−0.47, 0.52)] (SUCRA, 74.1%) was probably the most effective intervention for improving wrist joint motor function amongst subjects with strokes, followed by RR [(SMD = 0.06, 95%CI = (−0.35, 0.46)] (SUCRA, 70.1%); RT [(SMD = 0.11, 95%CI = (−0.29, 0.51)] (SUCRA, 63.8%); BCI [(SMD = 0.02, 95%CI = (−0.52, 0.55)] (SUCRA, 40.8%); VR [(SMD = −0.001, 95%CI = (−0.41, 0.41)] (SUCRA, 38.5%); CT [(SMD = 0.05, 95%CI = (−0.33, 0.44)] (SUCRA, 32.5%) and IR (SUCRA, 30.4%).

**Table 5:** Network analysis results of the FMA - UE - distal.

**RT+VR**						
0.03 (−0.47, 0.52)	RR					
0.08 (−0.32, 0.48)	0.06 (−0.35, 0.46)	RT				
0.19 (−0.29, 0.68)	0.17 (−0.32, 0.66)	0.11 (−0.29, 0.51)	BCI			
0.21 (−0.33, 0.74)	0.18 (−0.35, 0.72)	0.13 (−0.33, 0.58)	0.02 (−0.52, 0.55)	VR		
0.21 (−0.14, 0.55)	0.18 (−0.17, 0.53)	0.13 (−0.08, 0.33)	0.01 (−0.33, 0.36)	−0.00 (−0.41, 0.41)	CT	
0.26 (−0.26, 0.78)	0.24 (−0.29, 0.76)	0.18 (−0.26, 0.62)	0.07 (−0.45, 0.59)	0.05 (−0.51, 0.61)	0.05 (−0.33, 0.44)	IR

#### Action research arm test

A total of 22 studies were included in the ARAT comparison (60, 81, 82, 131, 133, 139, 142, 144, 149). As the network evidence map in this study did not form a closed loop, inconsistency tests could not be performed (148). However, *P* > 0.05 in the consistency test indicated excellent consistency and stability of the studies.

The NMA results showed significant differences in all interventions compared to CT, suggesting RT + VR [(SMD = 0.73, 95%CI = (0.20, 1.26)], VR [(SMD = 0.73, 95%CI = (0.14, 1.32)], BCI [(SMD = 0.78, 95%CI = (0.25, 1.31)], RT [(SMD = 0.93, 95%CI = (0.17, 1.70)], IR [(SMD = 0.92, 95%CI = (0.36, 1.48)] and RR [(SMD = 0.91, 95%CI = (0.44, 1.39)] were effective in improving ARAT function in subject with stroke (see Table 6). Figure 4E shows the SUCRA rankings for all treatments. Based on the results of the SUCRA analysis, RT + VR (SUCRA, 99.6%) was the most effective intervention for improving hand function amongst subjects with strokes, followed by VR (SUCRA, 60.9%), BCI (SUCRA, 57.7%), RT (SUCRA, 51.9%), IR (SUCRA, 30.1%), RR (SUCRA, 27.0%) and CT (SUCRA, 22.8%).

**Table 6:** Network analysis results of the ARAT.

**RT+VR**						
0.73 (0.20, 1.26)	VR					
0.73 (0.14, 1.32)	0.00 (−0.41, 0.42)	BCI				
0.78 (0.25, 1.31)	0.05 (−0.27, 0.37)	0.05 (−0.36, 0.46)	RT			
0.93 (0.17, 1.70)	0.20 (−0.44, 0.85)	0.20 (−0.49, 0.89)	0.15 (−0.49, 0.79)	IR		
0.92 (0.36, 1.48)	0.19 (−0.18, 0.56)	0.19 (−0.27, 0.64)	0.14 (−0.23, 0.51)	−0.01 (−0.68, 0.66)	RR	
0.91 (0.44, 1.39)	0.18 (−0.04, 0.41)	0.18 (−0.16, 0.53)	0.13 (−0.09, 0.36)	−0.02 (−0.62, 0.58)	−0.01 (−0.30, 0.29)	CT

### Presence of adverse effects

A network meta-analysis of adverse reactions could not be completed further as all included studies did not report adverse reactions.

### Publication bias and consistency assessment

**Figure 5:**
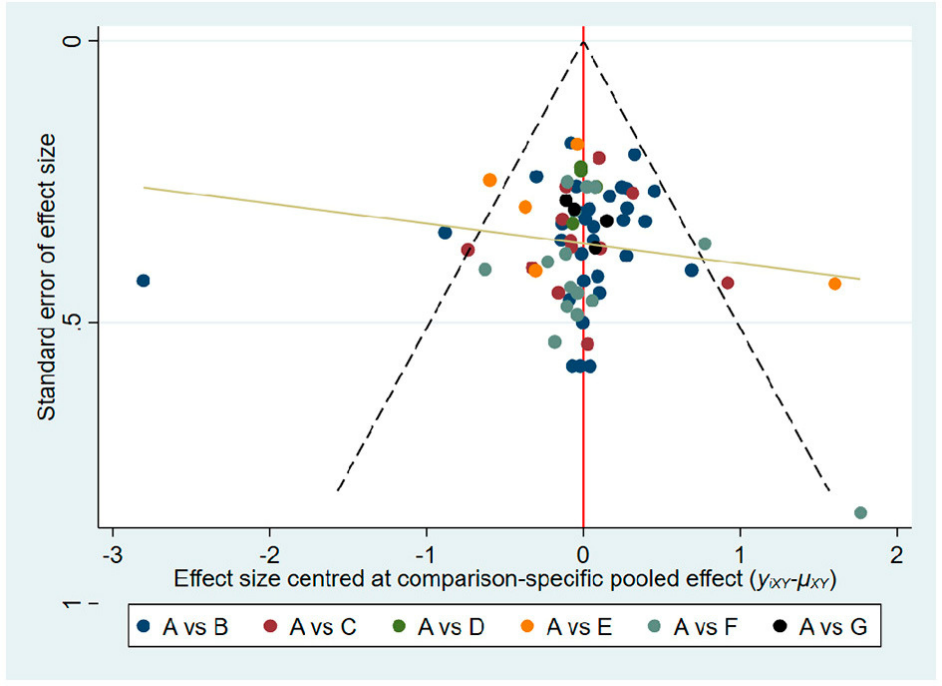
Comparison-adjusted funnel plots. FMA-UE total. The fuller the area under the curve, the more effective it is. A, conventional training (CT); B, Robot training (RT); C, Brain-computer interface (BCI); D, Remote rehabilitation (RR); E, Intelligent rehabilitation (IR); F, Virtual reality (VR); G, Robot training + virtual reality (RT+VR).

We constructed a comparative corrected funnel plot of the main results of FMA-UE-Total for evaluation *via* Stata/MP 16.0. Figure 5 shows the funnel plots show a symmetrical distribution, indicating limited publication bias in this study.

## Discussion

We conducted a systematic evaluation by NMA analysis, including 101 studies involving 4,702 subjects. The results of the NMA analysis showed that, in ARAT, there were significant differences in direct and indirect comparisons of RT + VR with each of the other interventions. In the FMA-UE-Proximal, there were significant differences in direct comparisons between RT + VR and RT vs. CT. Meanwhile, there were no significant differences between direct and indirect comparisons for each of the other interventions. However, in FMA-UE-Total, FMA-UE-Distal and MBI, there were no significant differences in direct and indirect comparisons between the interventions. Overall, there were no significant adverse effects in any of the studies, indicating the strong reliability and safety of the results.

Our NMA analyses may provide new and valuable insights into using different modalities of AI technology in the functional rehabilitation of the upper limb among subjects with strokes. We believe these analyses may be seen as complementary to previous systematic reviews on this topic.

In this study, we compared the effects of different AI techniques on FMA-UE-Proximal amongst subjects with strokes. The analysis showed that RT + VR [SMD = 0.26; 95% CI (−0.26, 0.78)] was the best treatment for improving overall outcomes in improving wrist and shoulder joint motor function in the upper limbs of subjects with strokes. RT + VR (SUCRA = 84.8%) was also the most effective treatment, according to the SUCRA results. Our NMA results also found a significant difference between RT + VR and RT in improving wrist joint motion amongst subjects with strokes compared with CT. In contrast, a single RCT by Chen and Jiang showed no significant difference between RT + VR in improving wrist motion amongst subjects with strokes (113, 150). Chen and Jiang further mentioned that, due to the absence of other forms of hand function training with RT + VR, the upper limb RT device often left the wrist and hand in a relatively fixed position compared with conventional exercise therapy. The improvement in hand function was not significant when compared with conventional rehabilitation. To some degree, this is inconsistent with the findings of this study. Some studies using RT technology to observe its effect on the function of the upper limb wrist and shoulder elbow joint in subjects with stroke have shown that upper limb RT can effectively improve the wrist and shoulder elbow joint function of the upper limb of subjects with stroke (64). Another large study showed that VR technology can also effectively improve the wrist motor function of the upper limbs of subjects with stroke (151). This also indirectly confirms that the use of RT combined with VR may be more advantageous, such as the NMA results indicated in this study, which showed that RT + VR is the best treatment to improve the function of upper limbs, shoulders, elbows and wrists in subjects with strokes. At the same time, the search at home and abroad found that most scholars did not separately evaluate the three sub-terms of shoulder joint, wrist joint and elbow joint in FMA-UE to observe the effect of RT, VR and RT + VR technology on the motor function of the above three joints in subjects with stroke. The meta-analysis did not classify the three sub-terms of FMA-UE, so it was not possible to observe the effects of RT and VR technology on the motor function of the above three joints in subjects with stroke by meta-synthesis (37, 152).

The present study, in contrast, is based on a multiple comparison NMA analysis and provides a summary of previous studies. Therefore, the results of this study may be somewhat more convincing than the above studies. Similarly, RT significantly improved wrist motor function in the upper limbs of subjects with strokes, consistent with the findings from Kwon et al. (111). Moreover, the results combined with the NMA analysis further suggest that RT + VR [SMD = 0.43; 95% CI (0.01, 0.85)] may be the optimal treatment in terms of improving wrist motor function amongst subject with strokes. On the one hand, our NMA also found that direct and indirect comparisons between studies showed no significant differences in the FMA-UE-Total and FMA-UE-Distal comparisons. In FMA-UE-Distal, IR ranked first out of seven different AI techniques (SUCRA = 70.5%), reflecting that it was the optimal treatment. In the FMA-UE-Total, RT + VR [SMD = 0.26; 95% CI (−0.07, 0.60)] ranked first out of seven different AI techniques (SUCRA = 74.1%), reflecting that it was the optimal treatment. We speculate that the reason for this lack of significant difference may be related to the inconsistent duration of disease (acute, sub-acute and chronic), varying age ranges, and inconsistent Brunnstrom motor function staging and balance in the subjects included in the original study (60, 72, 86, 88, 109).

The ARAT assesses changes in limb function, including the ability to manage objects with different physical characteristics. It is a valid and applicable assessment of changes in upper limb motor function following the onset of a stroke. It involves 19 items divided into four sub-scales: grip, grip strength, pinch and gross motor (153, 154). In this study, our NMA results suggest that RT + VR [SMD = 0.91; 95% CI (0.44, 1.39)] was most effective in improving ARAT in subjects with upper limb dysfunction after a stroke. According to the SUCRA results, RT + VR (SUCRA = 99.6%) was the most effective treatment. Simultaneously, there was a significant difference between RT + VR in improving the motor function of the upper limbs of ARAT amongst subjects with strokes compared with other interventions. However, some high-quality meta-analyses and multi-center RCTs have provided mixed conclusions.

One of the Cochrane meta-analyses showed a significant improvement in upper extremity ARAT function in subjects with stroke who received an upper extremity RT intervention, with significant changes in arm function and no significant difference in arm strength (155). A multi-center large RCT from Lancet also showed that upper extremity RT training was ineffective in improving upper limb ARAT function in subjects with stroke (156). Although there was no statistically significant difference in the results, there was a clear trend toward improvement at 3 and 6 months, with the authors suggesting that the reason for the absence of a difference may be related to wear and tear on the upper extremity RT training apparatus, participant adherence, and attrition rates (156). A multi-center study of VR came to the same conclusion as Lancet, showing that VR did not show significant between-group differences in improving ARAT in subjects with strokes compared with CT (157). Meanwhile, the combined use of VR technology and the upper limb rehabilitation robot allows the two to complement each other, thus effectively improving the ARAT motor function of the upper limbs amongst subjects with strokes (82). In contrast, this study is based on multiple comparative NMA studies, summarizing previous studies' shortcomings through direct and indirect comparisons. Therefore, the reliability of the results of this study is somewhat convincing.

The MBI consists of 10 items, including eating, bathing, grooming, dressing, stooling, urinating, toileting, transferring, walking and ascending and descending stairs and is often used to assess basic ADLs amongst subjects with strokes (96, 158). The results of the NMA by Li et al. (36) found that different modalities of BCI improved upper limb motor function and ADLs amongst subjects with strokes, with BCI and an FES as the driving device having the best effect (36). Our NMA analysis found that direct and indirect comparisons between studies showed no significant differences in improving the quality of daily life amongst subjects with strokes. At the same time, BCI [SMD = 0.18; 95% CI (−0.03, 0.39)] ranked best among seven different AI techniques (SUCRA = 73.6), reflecting that it was the most effective treatment. In conjunction with the study by Li et al., the BCI technique effectively improved the ability of subjects with strokes to perform daily living activities (36). Their findings are not consistent with those of our NMA study. We speculate that this may be related to the age, duration of disease, location of symptoms and functional recovery of the subjects included in the study (95, 159, 160).

This study provides an innovative, systematic integration of various AI rehabilitation techniques for direct and indirect comparison to establish which AI rehabilitation techniques were most effective in improving upper limb function in subjects with stroke. Based on clinical considerations, this study concluded that RT + VR had a significant advantage in improving shoulder and wrist joint motor function and ARAT in subjects with stroke, and IR and BCI had a significant advantage in improving upper limb motor function and MBI in FMA. Therefore, based on the evidence from this study, RT + VR, IR and BCI techniques can be recommended as the preferred treatment method for upper limb functional rehabilitation in subjects with stroke, which also provides evidence-based information for using and promoting AI rehabilitation techniques in clinical practice. Follow-up studies should provide more precise and personalized treatment protocols based on key characteristics of subjects with stroke, such as the severity of a stroke and the degree of upper limb impairment, as well as the intensity, frequency and duration of treatment, depending on their different clinical characteristics and degree of impairment. In terms of methodology, researchers also need to better describe interventions (both tailored and individualized) and ensure that the implementation and delivery of interventions are accurately documented, with attention to symptom reduction, independence and function. There should also be reporting on barriers to implementation and measuring the potential impact and harm of AI technologies.

## Strengths and limitations

First, our study included 101 studies and 4,702 patients, indicating a large sample size. Moreover, we involved seven treatment interventions and assessed the impact of the interventions in six ways to provide more comprehensive evidence-based recommendations. Second, most of the systematic reviews and meta-analyses of AI rehabilitation have assessed the effects of single RT, VR and BCI on upper limb function amongst subjects with strokes, with only one network meta-analysis reporting a study of different types of upper limb RT on upper limb function amongst subjects with strokes. We conducted the first NMA of different modalities of AI on upper limb function amongst subjects with strokes, providing the initial basis for further detailed studies in this area (39). This study also had limitations, including the following: (1) Many studies did not specifically report on randomization methods, allocation concealment and reliability of outcomes. The different treatment durations, frequencies and protocols included in the studies may have increased clinical heterogeneity. (2) Most original studies used semi-quantitative scales to assess shoulder-elbow and wrist joint motor function and total upper limb motor function scores and ADLs amongst subjects with strokes and did not use more objective and quantitative indicators. In subsequent studies, we must further use a combination of subjective and objective indicators to assess the improvement in their overall function. (3) We have created network diagrams that clearly illustrate the direct comparisons made in this domain; however, there were no closed loops in the network geometry. This led to our analysis not being an NMA or multiple treatment comparison (MTC) in the strictest sense but rather an adjusted indirect treatment comparison (ITC) belonging to the genus NMA.

## Conclusion

The results of our NMA and SUCRA rankings suggest RT + VR appears to have an advantage over other interventions in improving upper limb motor function amongst subjects with stroke with FMA-UE-Proximal, FMA-UE-Distal and ARAT. Similarly, IR had shown the most significant advantage over other interventions in improving the FMA-UE-Total upper limb motor function score of subjects with stroke. The BCI also had the most significant advantage in improving their MBI daily living ability. In addition, it is worth noting that future studies should consider and report key patient characteristics such as stroke severity and degree of upper limb impairment, as well as treatment intensity, frequency and duration. Future meta-analyses should consider sub-group analyses based on the duration of the subject's illness and intervention and their gender to comprehensively explore the impact of different AI modalities and techniques on subjects with stroke from different populations.

## Data availability statement

The original contributions presented in the study are included in the article/Supplementary material, further inquiries can be directed to the corresponding author.

## Author contributions

Conceptualization: YZ and CW. Methodology and software: CW and JL. Validation, investigation, and data management: YZ and LZ. Formal analysis and visualization: LZ. Resources: YZ. Writing—original draft preparation: YZ, CW, and PZ. Writing—review and editing: YZ, CW, JL, LZ, and PZ. Supervision and funding acquisition: PZ. Project management: YZ and PZ. All authors contributed to the article and approved the submitted version.
