# Supplementary material for: Effect of different modalities of artificial intelligence rehabilitation techniques on patients with upper limb dysfunction after stroke—A network meta-analysis of randomized controlled trials
Source: Front Neurol. 2023 Apr 17;14:1125172. doi: 10.3389/fneur.2023.1125172 (PMC10150552; doi:10.3389/fneur.2023.1125172)

**Supplementary Table S1.** Search strategy (take PubMed as an example)

#1 ((((((((rehabilitation) OR (routine rehabilitation)) OR (rehabilitation robots)) OR (robot)) OR (brain-computer interface)) OR (remote rehabilitation)) OR (intelligent rehabilitation)) OR (virtual reality)) OR (VR)

#2 (((((((stroke) OR (cerebral apoplexy)) OR (cerebral stroke)) OR (ischemic stroke)) OR (stroke recovery period)) OR (convalescent cerebral apoplexy)) OR (stroke convalescence)) OR (stroke recovery)

#3 ((((((((upper limb function) OR (limb function)) OR (function of upper extremities)) OR (limbs motor function)) OR (upper-limb motor function)) OR (hand function)) OR (hand functional)) OR (manual ability)) OR (function of the hands)

#4 #1 AND #2 AND #3

**Search strategies for other English and Chinese databases are as follows**

1. CNKI

检索式：((主题=康复) OR (主题=常规康复) OR (主题=机器人) OR (主题=康复机器人) OR (主题=脑机接口) OR (主题=远程康复) OR (主题=智能康复) OR (主题=虚拟现实) OR (主题=VR)) AND ((主题=脑卒中) OR (主题=脑梗) OR (主题=脑卒中恢复期)) AND ((主题=上肢功能) OR (主题=手功能))

1. 维普

检索式：((M=康复) OR (U=常规康复) OR (U=机器人) OR (U=康复机器人) OR (U=脑机接口) OR (U=远程康复) OR (U=智能康复) OR (U=虚拟现实) OR (U=VR)) AND ((M=脑卒中) OR (M=脑梗) OR (M=脑卒中恢复期)) AND ((M=上肢功能) OR (M=手功能))

1. 万方

检索式：(主题:(康复) or 全部:(常规康复) or 全部:(机器人) or 全部:(康复机器人) or 全部:(脑机接口) or 全部:(远程康复) or 全部:(智能康复) or 全部:(虚拟现实) or 全部:(VR)) and (主题:(脑卒中) or 主题:(脑梗) or 主题:(脑卒中恢复期)) and (主题:(上肢功能) or 主题:(手功能))

1. the Cochrane Library

Search Strategy：

#1: (rehabilitation) OR (routine rehabilitation) OR (rehabilitation robots) OR (robot) OR (brain-computer interface) OR (remote rehabilitation) OR (intelligent rehabilitation) OR (virtual reality) OR (VR)

#2: (stroke) OR (cerebral apoplexy) OR (cerebral stroke) OR (ischemic stroke) OR (stroke recovery period) OR (convalescent cerebral apoplexy) OR (stroke convalescence) OR (stroke recovery)

#3: (upper limb function) OR (limb function) OR (function of upper extremities) OR (limbs motor function) OR (upper-limb motor function) OR (hand function) OR (hand functional) OR (manual ability) OR (function of the hands)

#4: #1 AND #2 AND #3

1. Embase

Search Strategy：

#1: rehabilitation'/exp OR rehabilitation OR (routine AND ('rehabilitation'/exp OR rehabilitation)) OR (('rehabilitation'/exp OR rehabilitation) AND robots) OR 'robot'/exp OR robot OR ('brain computer' AND ('interface'/exp OR interface)) OR (remote AND ('rehabilitation'/exp OR rehabilitation)) OR (intelligent AND ('rehabilitation'/exp OR rehabilitation)) OR (virtual AND ('reality'/exp OR reality)) OR vr

#2: stroke OR (cerebral AND apoplexy) OR (cerebral AND stroke) OR (ischemic AND stroke) OR (stroke AND recovery AND period) OR (convalescent AND cerebral AND apoplexy) OR (stroke AND convalescence) OR (stroke AND recovery)

#3: upper AND limb AND function OR (limb AND function) OR (function AND of AND upper AND extremities) OR (limbs AND motor AND function) OR ('upper limb' AND motor AND function) OR (hand AND function) OR (hand AND functional) OR (manual AND ability) OR (function AND of AND the AND hands)

#4: #1 AND #2 AND #3 AND 'randomized controlled trial'/de

1. Web of science

Search Strategy：

#1: (rehabilitation) OR (routine rehabilitation) OR (rehabilitation robots) OR (robot) OR (brain-computer interface) OR (remote rehabilitation) OR (intelligent rehabilitation) OR (virtual reality) OR (VR)

#2: (stroke) OR (cerebral apoplexy) OR (cerebral stroke) OR (ischemic stroke) OR (stroke recovery period) OR (convalescent cerebral apoplexy) OR (stroke convalescence) OR (stroke recovery)

#3: (upper limb function) OR (limb function) OR (function of upper extremities) OR (limbs motor function) OR (upper-limb motor function) OR (hand function) OR (hand functional) OR (manual ability) OR (function of the hands)

#4: #1 AND #2 AND #3

**Supplementary Table S2**. Description of the basic characteristics of the included studies.

| Study | Location | Duration of intervention | Type | Group | n | Sex(M/F) | Age(years) | Ending indicators |
| --- | --- | --- | --- | --- | --- | --- | --- | --- |
| Lili Su et al.,2022 | China | 4 weeks | Sub-acute stroke | EG:RT+CT | 60 | 14/16 | 65.53±5.46 | FMA-UE, FMA-D, FMA-P, MBI |
|  |  |  |  | CG:CT |  | 15/15 | 64.97±4.88 |  |
| Sahel et al.,2021 | Turkey | 12 weeks | Chronic stroke | EG:RT+CT | 37 | 14/3 | 50.94±17.20 | FMA-UE |
|  |  |  |  | CG:CT |  | 14/6 | 55.75±11.61 |  |
| Patrizio et al.,2014 | Italy | 6 weeks | Sub-acute stroke | EG:RT | 53 | 11/15 | 67.7(65.8-77.0) | FMA-UE |
|  |  |  |  | CG:CT |  | 11/16 | 67.7(69.0-78.0) |  |
| Yan He et al.,2021 | China | 4 weeks | Stroke | EG1:RT+CT | 60 | 26/4 | 57.67±12.98 | FMA-UE, MBI |
|  |  |  |  | CG:CT |  | 26/4 | 57.53±14.61 |  |
| Charles et al.,2011 | USA | 6 months | Stroke | EG1:RT-Lo | 54 | 9/10 | 62.5 ± 2.0 | FMA -UE, |
|  |  |  |  | EG2:RT-Hi |  | 9/8 | 58.6 ± 2.3 |  |
|  |  |  |  | CG:CT |  | 5/13 | 68.1 ± 3.3 |  |
| Rodrigo et al.,2011 | Brazil | 8 weeks | Chronic stroke | EG:RT | 12 | 5/1 | 42.83±14.04 | FMA-UE, FMA-D, FMA-P |
|  |  |  |  | CG:CT |  | 5/1 | 52.67±17.84 |  |
| Sarah J et al.,2009 | USA | 6 months | Chronic stroke | EG:RT | 28 | 7/7 | 54.2 ± 11.9 | FMA-UE, MBI |
|  |  |  |  | CG:CT |  | 11/3 | 56.4 ± 12.8 |  |
| Yu-wei Hsieh et al.,2011 | Taiwan | 4 weeks | Chronic stroke | EG1:HI-RT | 18 | 4/2 | 56.04 ± 13.07 | FMA -UE, |
|  |  |  |  | EG2:LI-RT |  | 4/2 | 52.45 ± 1.98 |  |
|  |  |  |  | CG:CT |  | 5/1 | 54.00 ± 8.05 |  |
| Stephen et al.,2012 | USA | 8 weeks | Stroke | EG:RT | 16 | 3/5 | 59.0 ± 12.9 | FMA-UE, MBI |
|  |  |  |  | CG:CT |  | 8/0 | 58.5 ± 9.5 |  |
| Annick et al.,2014 | Netherlands | 6 months | Chronic stroke | EG:RT | 22 | 8/3 | 61.8 ± 6.8 | FMA-UE, ARAT |
|  |  |  |  | CG:CT |  | 8/3 | 56.8 ± 6.4 |  |
| Aamani et al.,2021 | Singapore | 6 weeks | Sub-acute stroke | EG:RT | 44 | 11/11 | 56.32 ± 10.37 | FMA-UE, ARAT |
|  |  |  |  | CG:CT |  | 14/8 | 54.59 ± 10.92 |  |
| Haiyan Zhang et al.,2019 | China | 4 weeks | Stroke | EG:RT+CT | 40 | 14/6 | 67.3 ± 6.0 | FMA-UE, MBI, |
|  |  |  |  | CG:CT+OT |  | 12/8 | 66.4 ± 4.4 |  |
| Changcheng Sun et al.,2018 | China | 4 weeks | Stroke | EG:RT+CT | 70 | 21/17 | 59.11 ± 9.99 | FMA-UE, MBI |
|  |  |  |  | CG:CT |  | 17/15 | 58.06 ± 10.70 |  |
| Tijana et al.,2017 | Serbia | 3 weeks | Stroke | EG:RT | 26 | 12/1 | 56.5 ± 7.4 | FMA-UE, MBI |
|  |  |  |  | CG:CT |  | 9/4 | 58.3 ± 5.2 |  |
| Susan et al.,2019 | USA | 12 weeks | Chronic stroke | EG:RT+CT | 45 | 15/8 | 56.4 ± 12.7 | FMA-UE, FMA-D, FMA-P |
|  |  |  |  | CG:CT+TTT |  | 14/8 | 55.7 ± 10.2 |  |
| Hong Fan et al.,2016 | China | 12 weeks | Acute-stroke | EG:RT+CT | 100 | 29/21 | 64.46 ± 8.81 | FMA-UE, FMA-D, FMA-P,MBI |
|  |  |  |  | CG:CT |  | 30/20 | 68.00 ± 8.81 |  |
| Kyeong et al.,2016 | Korea | 2 weeks | Stroke | EG:RT | 44 | 15/7 | 50.27±11.11 | MBI |
|  |  |  |  | CG:CT |  | 14/8 | 52.32±8.66 |  |
| Jorge et al.,2017 | Italy | 3 weeks | Stroke | EG:RT+CT | 32 | 11/5 | NA | MBI |
|  |  |  |  | CG:CT |  | 10/6 | NA |  |
| Chao Zhang et al.,2016 | China | 4 weeks | Stroke | EG:RT+CT | 12 | 4/2 | 35.5±9.0 | FMA-UE |
|  |  |  |  | CG:CT |  | 5/1 | 47.0±10.0 |  |
| Xiufang Zhang et al.,2016 | China | 1 month | Stroke | EG:RT+CT | 40 | 12/8 | 53.2 ± 9.1 | FMA-UE, MBI |
|  |  |  |  | CG:CT |  | 11/9 | 52.9 ± 8.6 |  |
| Bin He et al.,2016 | China | 12 weeks | Acute-stroke | EG:RT+CT | 46 | 16/7 | 55.82 ± 11.25 | FMA-UE, MBI |
|  |  |  |  | CG:CT+TTT |  | 15/8 | 54.37 ± 11.02 |  |
| Neha et al.,2021 | India | 4 weeks | Chronic stroke | EG:RT | 23 | NA | 41.1 ± 12.8 | FMA-UE, FMA-D, FMA-P, MBI |
|  |  |  |  | CG:CT |  | NA | 42.7 ± 9.3 |  |
| Shangrong Jiang et al.,2021 | China | 2 weeks | Sub-acute stroke | EG:RT | 45 | 9/14 | 62.43 ± 11.29 | FMA-UE, MBI |
|  |  |  |  | CG:CT |  | 7/15 | 66 ± 11.51 |  |
| Marialuisa et al.,2019 | Italy | 12 weeks | Chronic stroke | EG:RT | 32 | 12/4 | 59.31 ± 14.40 | FMA -UE |
|  |  |  |  | CG:CT |  | 10/6 | 59.13 ± 14.97 |  |
| Dehem et al.,2019 | Belgium | 6 months | Stroke | EG:RT | 45 | 11/12 | 67.1 ± 11.1 | FMA-UE, |
|  |  |  |  | CG:CT |  | 10/12 | 68.6 ± 19.1 |  |
| Irene et al.,2020 | Italy | 3 months | Sub-acute stroke | EG:RT | 224 | 63/48 | 69.5 ± 10.9 | FMA-UE, MBI |
|  |  |  |  | CG:CT |  | 64/49 | 68.5 ± 11.5 |  |
| Quan Xu et al.,2020 | China | 6 weeks | Sub-acute stroke | EG:RT | 40 | 15/5 | 62.2 ± 10.1 | FMA-UE,MBI |
|  |  |  |  | CG:OT |  | 14/6 | 60.7 ± 10.6 |  |
| Yanhuan Huang et al.,2018 | Hong Kong | 5 weeks | Chronic stroke | EG:Clinic-RT | 32 | 8/8 | 53.50 ± 13.08 | FMA-UE, ARAT,FMA-D,FMA-P |
|  |  |  |  | CG:Lab-RT |  | 12/4 | 53.06 ± 10.27 |  |
| Evan et al.,2015 | Hong Kong | 6 months | Chronic stroke | EG:RT | 19 | 7/2 | 50.7 ± 9.0 | ARAT, FMA-UE,FMA-D,FMA-P |
|  |  |  |  | CG:CT |  | 7/3 | 55.1 ± 10.6 |  |
| Ilaria et al.,2020 | Italy | 3 months | Chronic stroke | EG:RT | 38 | 9/10 | 67.0 (58.0–70.0) | FMA-UE,FMA-D,FMA-P |
|  |  |  |  | CG:CT |  | 9/10 | 59.0 (46.0–69.0) |  |
| Stephanie et al.,2019 | Korea | 4 weeks | Chronic stroke | EG1:EXO-RT | 38 | 15/4 | 49.47 ± 10.88 | FMA-UE, MBI |
|  |  |  |  | CG:EE-RT |  | 11/8 | 54.00 ± 10.01 |  |
| Ching-yi Wu et al.,2012 | Taiwan | 4 weeks | Chronic stroke | EG1:RBAT | 42 | 10/4 | 55.13 ± 12.72 | FMA-UE,FMA-D,FMA-P,MBI |
|  |  |  |  | EG2:TBAT |  | 12/2 | 57.04 ± 8.78 |  |
|  |  |  |  | CG:CT |  | 10/4 | 51.30 ± 6.23 |  |
| Ehab Mohamed et al.,2022 | Saudi Arabia | 12 weeks | stroke | EG:VRT+RT+CT | 36 | NA | NA | ARAT |
|  |  |  |  | CG:CT |  |  |  |  |
| Linyong Hu et al.,2022 | China | 4 weeks | stroke | EG1:VRT+CT | 65 | 14/8 | 56.64±11.37 | FMA-UE, MBI, ARAT |
|  |  |  |  | EG2:RT+CT |  | 11/11 | 59.78±11.13 |  |
|  |  |  |  | EG3:VRT+RT+CT |  | 14/7 | 57.89±11.88 |  |
| Mingrong Chen et al.,2021 | China | 2 weeks | stroke | EG:VRT+RT+CT | 30 | 12/3 | 59.40±11.06 | FMA-UE, MBI |
|  |  |  |  | CG:CT |  | 7/8 | 63.60±10.04 |  |
| Yuanchun Wang et al.,2021 | China | 8 weeks | Chronic stroke | EG1 :RT+CT | 48 | 13/11 | 56.16±4.52 | FMA-UE, MBI |
|  |  |  |  | EG2:VRT+CT |  | 14/10 | 55.72±4.66 |  |
| Tereza et al.,2021 | Czech Republic | 3 weeks | Sub-acute stroke | EG :VRT | 50 | 14/11 | 66.56±12.26 | FMA-UE |
|  |  |  |  | CG:CT |  | 15/10 | 68.12±11.97 |  |
| Chen-Guang Zhao et al.,2022 | China | 4 weeks | Sub-acute stroke | EG :BCIT | 28 | 13/1 | 50.1±11.1 | FMA-UE,MBI |
|  |  |  |  | CG:CT |  | 12/2 | 56.16±11.5 |  |
| Lili Wang et al.,2022 | China | 4 weeks | stroke | EG:BCIT+CT | 40 | 9/11 | 69.05±5.79 | FMA-UE,MBI, ARAT |
|  |  |  |  | CG:CT |  | 12/8 | 67.25±4.78 |  |
| Su-Hyun et al.,2020 | Korea | 4 weeks | stroke | EG:BCIT+CT | 26 | 4/9 | 55.15±11.57 | FMA-UE, MBI, WMFT |
|  |  |  |  | CG:CT |  | 6/7 | 58.30±9.19 |  |
| Kai Keng Ang et al.,2014 | Singapore | 6 weeks | stroke | EG1:BCI-Manus | 26 | 9/2 | 48.5±13.5 | FMA-UE |
|  |  |  |  | EG2:Manus |  | 8/7 | 53.6±9.5 |  |
| Ying Xu et al.,2018 | China | 8 weeks | stroke | EG:BCI+CT | 32 | 15/1 | 72.42±8.56 | FMA-UE, MBI |
|  |  |  |  | CG:CT |  | 15/1 | 76.81±9.57 |  |
| Sijie Liang et al.,2020 | China | 4 weeks | stroke | EG:BCI+CT | 30 | 12/3 | 57.94±8.84 | FMA-UE, MBI |
|  |  |  |  | CG:CT |  | 9/6 | 50.06±13.46 |  |
| Mingfen Li et al.,2012 | China | 8 weeks | stroke | EG:BCI+CT | 14 | 5/2 | 66.29±4.89 | FMA-UE, ARAT |
|  |  |  |  | CG:CT |  | 5/2 | 60.00±6.30 |  |
| Xiangxian Wen et al.,2020 | China | 6 weeks | stroke | EG:BCI+CT | 94 | 22/25 | 58.6±2.7 | FMA-UE, MBI |
|  |  |  |  | CG:CT |  | 26/21 | 60.2±1.9 |  |
| Hai Ren et al.,2020 | China | 4 weeks | stroke | EG:BCI+CT | 60 | 18/12 | 41.77±8.65 | FMA-UE, MBI |
|  |  |  |  | CG:CT |  | 20/10 | 40.7±8.15 |  |
| Shugeng Chen et al.,2020 | China | 4 weeks | stroke | EG:BCI | 14 | 7/0 | 41.6±12.0 | FMA-UE |
|  |  |  |  | CG:CT |  | 5/2 | 52.0±11.1 |  |
| Alexander A et al., 2017 | American | 2 weeks | stroke | EG:BCI+CT | 74 | 34/21 | 58.0±12.59 | FMA-UE, ARAT |
|  |  |  |  | CG:CT |  | 14/5 | 58.0±11.11 |  |
| TaeHoon et al.,2015 | American | 4 weeks | stroke | EG:BCI+CT | 30 | 6/9 | 59.07±8.97 | FMA-UE, MBI |
|  |  |  |  | CG:CT |  | 6/9 | 59.93±9.79 |  |
| Masahito et al.,2013 | American | 2 weeks | stroke | EG:BCI+CT | 20 | 8/2 | NA | FMA-UE, ARAT |
|  |  |  |  | CG:CT |  | 4/6 | NA |  |
| Huaming Zhang et al.,2020 | China | 8 weeks | stroke | EG:BCIT+CT | 30 | 11/4 | 60.93±6.76 | FMA-UE, MBI |
|  |  |  |  | CG:CT |  | 11/4 | 57.87±8.61 |  |
| Hongling Wu et al.,2022 | China | 8 weeks | Chronic stroke | EG:RR+CT | 80 | 19/21 | 57.45±9.98 | FMA-UE, MBI |
|  |  |  |  | CG:CT |  | 27/13 | 61.45±9.83 |  |
| Jianqin Xue et al.,2020 | China | 8 weeks | Chronic stroke | EG:RR+CT | 60 | NA | NA | FMA-UE, MBI |
|  |  |  |  | CG:CT |  | NA | NA |  |
| Ran Wang et al.,2020 | China | 4 weeks | Chronic stroke | EG:RR+CT | 38 | 15/4 | 53.22±10.65 | FMA-UE, MBI |
|  |  |  |  | CG:CT |  | 14/5 | 53.05±14.83 |  |
| Fei Wang et al.,2018 | China | 48 weeks | stroke | EG:RR+CT | 60 | 24/6 | 58.0±12 | FMA-UE, MBI |
|  |  |  |  | CG:CT |  | 22/8 | 60.00±9 |  |
| Xiuming Gao et al.,2017 | China | 12 weeks | stroke | EG:RR+CT | 40 | 12/6 | 53.2±17.1 | FMA-UE, MBI |
|  |  |  |  | CG:CT |  | 14/8 | 52.2±14.1 |  |
| Jing Chen et al.,2017 | China | 24 weeks | stroke | EG:RR+CT | 54 | 18/9 | 66.52±12.08 | FMA-UE, MBI |
|  |  |  |  | CG:CT |  | 15/12 | 66.15±12.33 |  |
| Jing Chen et al.,2016 | China | 8 weeks | stroke | EG:RR | 44 | 26/6 | 65.3±13.2 | FMA-UE |
|  |  |  |  | CG:CT |  | 14/8 | 67.1±10.7 |  |
| Yan Jin et al.,2015 | China | 24 weeks | stroke | EG:RR | 100 | 31/19 | 66.50±11.45 | MBI |
|  |  |  |  | CG:CT |  | 36/14 | 66.7±11.76 |  |
| Kwan-Hwa Lin et al.,2014 | China | 4 weeks | stroke | EG:RR | 24 | 2/10 | 74.6±2.3 | MBI |
|  |  |  |  | CG:CT |  | 5/7 | 75.6±3.4 |  |
| Pakaratee et al.,2012 | Thailand | 24 weeks | stroke | EG:RR | 60 | 14/16 | 67±7 | MBI |
|  |  |  |  | CG:CT |  | 13/17 | 66±11 |  |
| Nor Shahizan et al.,2012 | Malaya | 12 weeks | stroke | EG:RR | 90 | 21/23 | 63.7±12 | MBI |
|  |  |  |  | CG:CT |  | 31/15 | 59.40±11 |  |
| Lamberto et al.,2009 | Venezia | 4 weeks | stroke | EG:RR | 36 | 11/7 | 66±7.9 | FMA-UE |
|  |  |  |  | CG:CT |  | 10/8 | 64.4±7.9 |  |
| Jiong Li et al.,2011 | China | 12 weeks | stroke | EG:RR | 101 | 28/23 | 65.69±11.32 | FMA-UE, MBI |
|  |  |  |  | CG:CT |  | 27/23 | 65.51±13.02 |  |
| Jae-Sung Kwon et al.,2012 | Korea | 4 weeks | stroke | EG:VR | 26 | 9/4 | 57.14±15.42 | FMA - UE, FMA - P, FMA - D, MBI |
|  |  |  |  | CG:CT |  | 5/8 | 57.92±12.32 |  |
| Jiawei Chen et al.,2020 | China | 48 weeks | stroke | EG:VR+RT | 49 | 19/4 | 64.31±6.11 | FMA - UE, FMA - P, FMA - D, MBI |
|  |  |  |  | CG:CT |  | 14/12 | 66.42±5.6 |  |
| Rongrong Jiang et al.,2017 | China | 2 weeks | stroke | EG:VRT+RT+CT | 40 | 9/11 | 63.15±11.79 | FMA - UE, FMA- P, FMA - D, MBI |
|  |  |  |  | CG:CT |  | 15/5 | 65.10±9.14 |  |
| Yi Wei et al.,2019 | China | 3 weeks | Stroke | EG:IR | 120 | 37/23 | 66.3±5.2 | FMA - UE |
|  |  |  |  | CG:CT |  | 35/25 | 65.7±5.4 |  |
| Man Wang et al.,2020 | China | 12 weeks | Stroke | EG:IR+CT | 110 | 27/28 | 64.23±5.95 | FMA - UE |
|  |  |  |  | CG:CT |  | 31/24 | 63.08±6.14 |  |
| Gerdienke B et al.,2015 | Netherlands | 6 weeks | Sub-acute stroke | EG:IR | 68 | 17/18 | 60.3±9.7 | FMA - UE |
|  |  |  |  | CG:CT |  | 14/19 | 58±11.4 |  |
| Kyeong Woo et al.,2016 | Korea | 2 weeks | Stroke | EG:IR+CT | 50 | 14/11 | 55.76±13.6 | MBI |
|  |  |  |  | CG:CT |  | 12/13 | 57.88±11.12 |  |
| Penelope A et al.,2015 | Australia | 2 weeks | Stroke | EG:IR | 41 | 13/8 | 59.9±13.8 | FMA - UE |
|  |  |  |  | CG:CT |  | 18/2 | 56.1±17 |  |
| Chueh-Ho et al.,2015 | China | 4 weeks | Chronic stroke | EG:TG | 33 | 12/4 | 52.63±10.49 | FMA - UE, FMA - P, FMA - D |
|  |  |  |  | CG:CT |  | 16/1 | 57.47±10.29 |  |
| Yasar et al.,2019 | Turkey | 6 weeks | Stroke | EG:VR+CT | 24 | 4/8 | 63.6±9.2 | FMA - UE |
|  |  |  |  | CG:CT |  | 5/7 | 63.6±7.1 |  |
| Ehab Mohamed et al.,2021 | Norway | 12 weeks | Chronic stroke | EG:VR+CT | 40 | 16/4 | 54.46±4.27 | ARAT |
|  |  |  |  | CG:CT |  | 15/5 | 53.32±5.13 |  |
| Naveed et al.,2021 | Pakistan | 6weeks | Stroke | EG:VR | 68 | 20/14 | 51.56±7.19 | FMA-UE |
|  |  |  |  | CG:CT |  | 14/20 | 51.35±5.78 |  |
| Zhenhua Jin et al.,2019 | China | 4 weeks | Stroke | EG:VR+CT | 60 | 16/14 | 70.31±3.81 | FMA-UE |
|  |  |  |  | CG:CT |  | 17/13 | 69.83±3.27 |  |
| Xiang Xiao et al.,2019 | China | 4 weeks | Sub-acute stroke | EG:VR | 35 | 10/6 | 56.12±9.01 | FMA-UE、MBI |
|  |  |  |  | CG:CT |  | 12/7 | 53.67±8.03 |  |
| Min Bo et al.,2017 | China | 4 weeks | Stroke | EG:VR+CT | 60 | 23/7 | 64.0±7.74 | FMA-UE、MBI |
|  |  |  |  | CG:CT |  | 25/5 | 62.4±9.77 |  |
| Xiaoxiao Han et al.,2016 | China | 2 weeks | Stroke | EG:VR | 30 | 10/5 | 61.4±8.1 | FMA-UE |
|  |  |  |  | CG:CT |  | 9/6 | 58.8±9.5 |  |
| Ran Tian et al.,2016 | China | 4 weeks | Stroke | EG:VR+CT | 60 | 21/9 | 57.4±11.34 | FMA-UE、MBI |
|  |  |  |  | CG:CT |  | 19/11 | 58.13±12.57 |  |
| Myung-mo et al.,2016 | Korea | 4 weeks | Stroke | EG:VR+CT | 10 | 3/2 | 65.2 ± 5.0 | FMA-UE |
|  |  |  |  | CG:CT |  | 2/3 | 66.2 ± 3.4 |  |
| Keng-He Kong et al.,2016 | Singapore | 3 weeks | Stroke | EG1:VR | 102 | 27/5 | 58.1 ± 9.1 | FMA-UE、ARAT |
|  |  |  |  | EG2:CT |  | 25/8 | 59.0 ± 13.6 |  |
|  |  |  |  | CG:CC |  | 25/12 | 55.8 ± 11.5 |  |
| Nahid et al.,2019 | Canada | 4 weeks | Stroke | EG:VR+CT | 18 | 5/4 | 42.2±9.5 | FMA-UE |
|  |  |  |  | CG:CT |  | 5/4 | 57.6±10.5 |  |
| Yoon-Hee et al.,2016 | Korea | 2 weeks | Ischemic stroke | EG:VR+CT | 24 | 7/5 | 61±15.2 | FMA-UE |
|  |  |  |  | CG:CT |  | 6/6 | 72.1±9.9 |  |
| Mina et al.,2019 | Korea | 4 weeks | Stroke | EG:VR+CT | 25 | 7/5 | 53.5±13.0 | FMA-UE, FMA-P, FMA-D, MBI |
|  |  |  |  | CG:CT |  | 8/5 | 51.5±16.7 |  |
| Iris Brunner et al.,2014 | Norway | 4 weeks | Stroke | EG:VR | 130 | 42/20 | 62±16.5 | ARAT |
|  |  |  |  | CG:CT |  | 35/23 | 62±11.5 |  |
| Jun Hwan et al.,2014 | Korea | 4 weeks | Sub-acute stroke | EG:VR | 20 | 5/5 | 64.30±10.3 | FMA-UE |
|  |  |  |  | CG:CT |  | 5/5 | 64.70±11.3 |  |
| Kelly O et al.,2014 | USA | 6 weeks | Stroke | EG:VR | 14 | 4/3 | 54±7 | ARAT |
|  |  |  |  | CG:CT |  | 5/2 | 59±6 |  |
| Pawel et al.,2014 | Italy | 4 weeks | Stroke | EG:VR | 44 | 14/9 | 63.1±9.5 | FMA-UE |
|  |  |  |  | CG:CT |  | 15/6 | 65.5±14.2 |  |
| Dong Jin et al.,2013 | Korea | 4 weeks | Stroke | EG:VR+CT | 24 | 5/7 | 58.33±10.17 | FMA-UE |
|  |  |  |  | CG:CT |  | 6/6 | 65.42±9.77 |  |
| Jae-Sung et al.,2012 | Korea | 4 weeks | Stroke | EG:VR+CT | 26 | 9/4 | 57.15±15.42 | FMA-UE、MBI |
|  |  |  |  | CG:CT |  | 5/8 | 57.92±12.32 |  |
| JH CTosbie et al.,2012 | UK | 3 weeks | Stroke | EG:VR | 18 | 5/4 | 56.1±14.5 | ARAT |
|  |  |  |  | CG:CT |  | 5/4 | 64.6±7.4 |  |
| Chanwai Yin et al.,2014 | Singapore | 2 weeks | Stroke | EG:VR+CT | 23 | 6/5 | 62±16.3 | FMA-UE、ARAT |
|  |  |  |  | CG:CT |  | 10/2 | 56±11.1 |  |
| Tiantian Zhou et al.,2019 | China | 12 weeks | Stroke | EG:RR+CT | 75 | 21/16 | 55.00±5.15 | ARAT,FMA-UE，MBI |
|  |  |  |  | CG:CT |  | 20/18 | 55.97±6.17 |  |
| Muhammed Nur et al.,2019 | Türkiye | 6 weeks | Ischemic stroke | EG:VR | 65 | 28/5 | 61.48 ± 10.92 | ARAT,FMA-UE，MBI |
|  |  |  |  | CG:CT |  | 23/9 | 59.75 ± 8.07 |  |
| Sharon M et al.,2016 | Netherlands | 6 weeks | Chronic stroke | EG:IR | 20 | 7/3 | 58±12.59 | ARAT |
|  |  |  |  | CG:CT |  | 3/7 | 62±11.85 |  |
| Steven L et al.,2015 | USA | 8 weeks | Stroke | EG:RR+CT | 99 | 31/17 | 54.7±12.2 | ARAT, FMA-UE,FMA-P, FMA-D |
|  |  |  |  | CG:CT |  | 25/26 | 59.1±14.1 |  |
| Ling Chen et al.,2022 | China | 2 weeks | Stroke | EG:VR | 36 | 10/8 | 57.8±8.4 | ARAT, FMA-UE |
|  |  |  |  | CG:CT |  | 10/8 | 58.4±9.3 |  |
| Debbie et al.,2016 | Israel | 5 weeks | Chronic stroke | EG:IR | 24 | 9/4 | 59.1±10.5 | ARAT |
|  |  |  |  | CG:CT |  | 6/5 | 64.9±6.9 |  |
| Qiuyang Qian et al.,2017 | China | 12 weeks | Sub-acute stroke | EG:RR | 24 | 9/5 | 54.6 ± 11.3 | ARAT, FMA-UE，FMA-P, FMA-D |
|  |  |  |  | CG:CT |  | 6/4 | 64.6 ± 3.43 |  |
| Barbara et al.,2008 | Netherlands | 4 weeks | Stroke | EG:RR+CT | 17 | 2/9 | 69±8 | ARAT |
|  |  |  |  | CG:CT |  | 4/1 | 71±7 |  |

Note: ARAT, action research arm test; AST, arm supporting training; BCI, brain-computer interface; CG, control group; CT, conventional rehabilitation; D, distal; RR, remote rehabilitation training; EG, experimental group; EE, end - efector; EXO, exoskeleton; FMA - UE, fugl-meyer assessment upper extremity; HI, higher intensity; IR, intelligent rehabilitation; LI, lower intensity; Lo, low dose; MBI, modified barthel index; mCIMT, modified constraint induced movement therapy; OT, occupational therapy; P, proximal; RBAT, robot-assised bilateral arm training; RT, robot treatment; RR, remote rehabilitation; TBAT, therapist-based arm training; TTT, transition-to-task therapy; VRT, virtual reality training; WMT, wii-based movement therapy.

**Supplementary Figure S1**. Risk of bias assessments for included studies


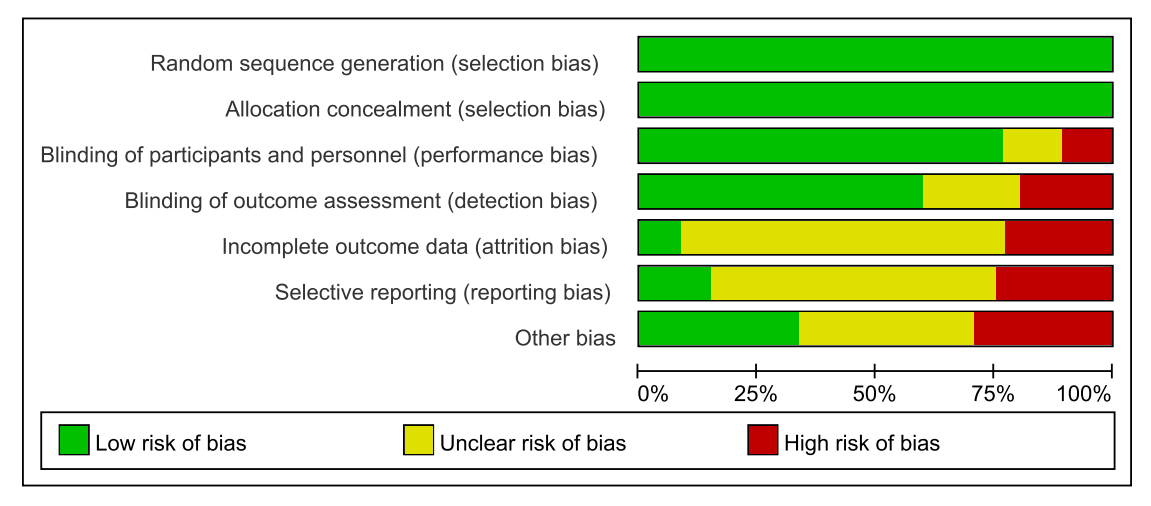


**Supplementary Figure S2.** The risk of bias evaluation


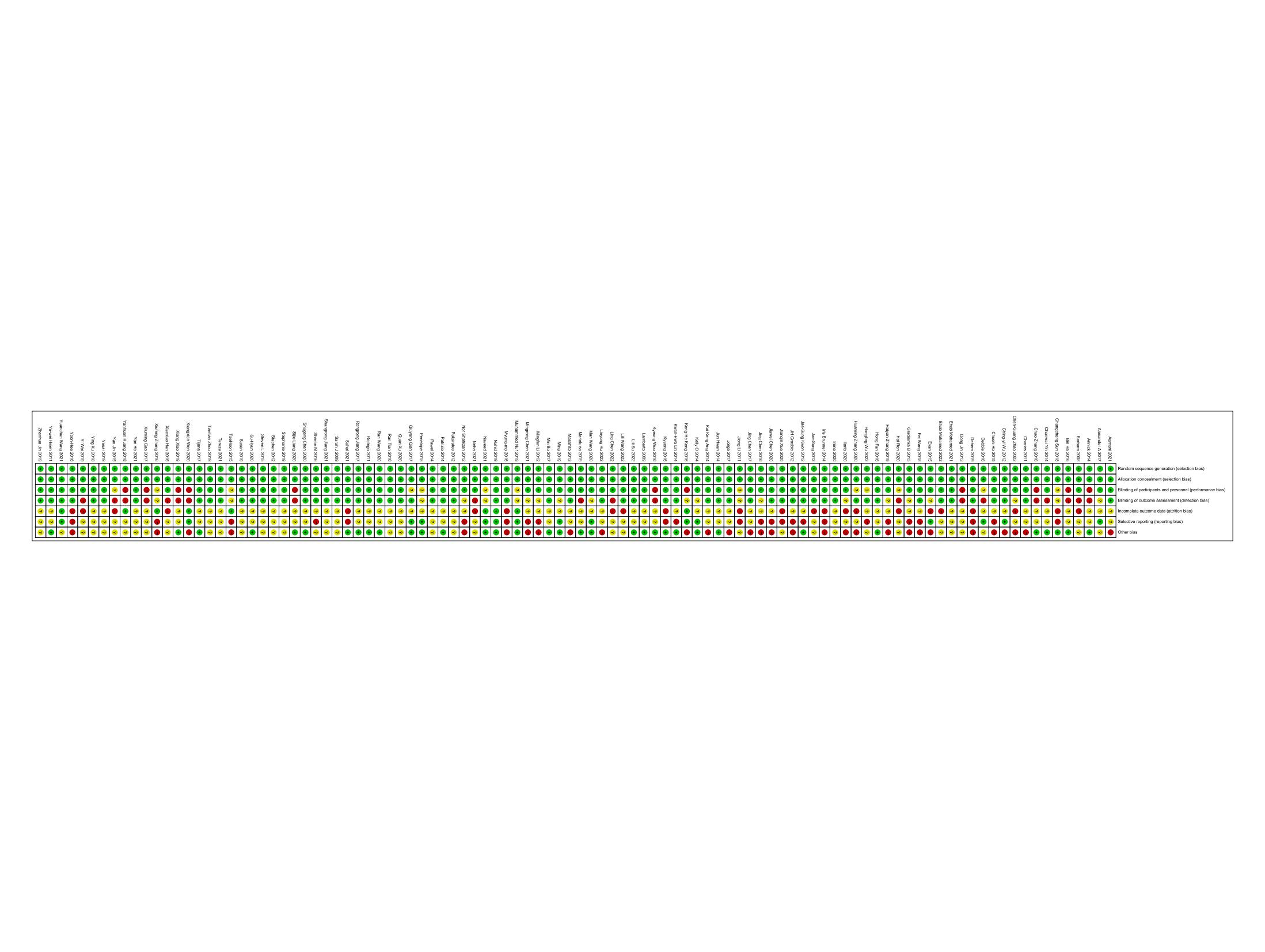


**Supplementary Figure S3.** Consistency assessment of network meta-analysis of FMA - UE - Total.

**Design consistency**


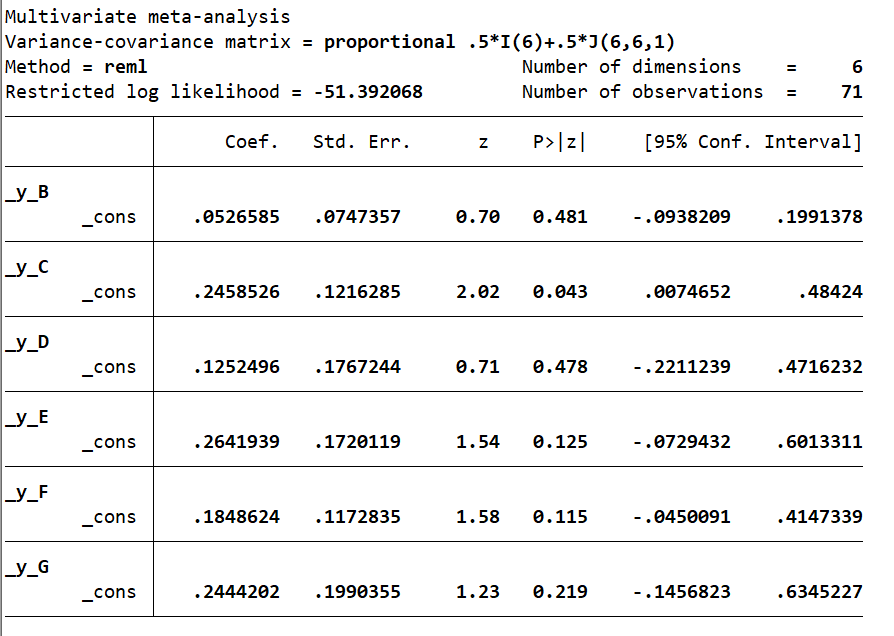


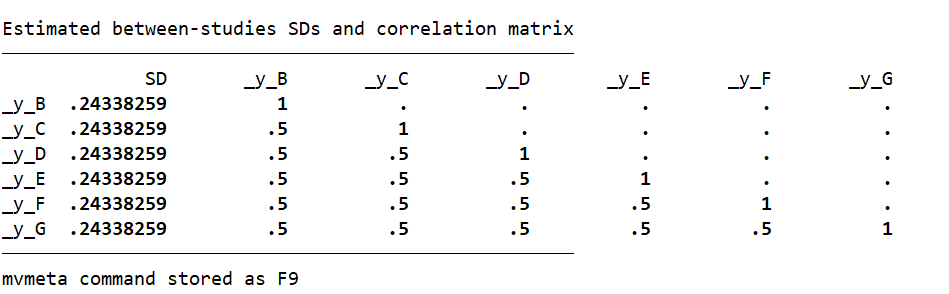


**Supplementary Figure S4.** Consistency assessment of network meta-analysis of MBI.

**Design consistency**


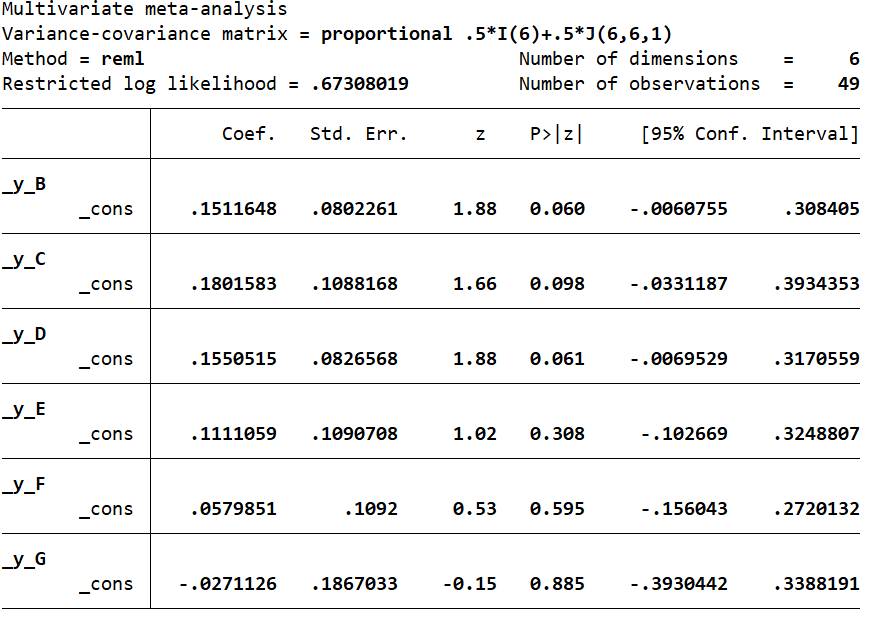


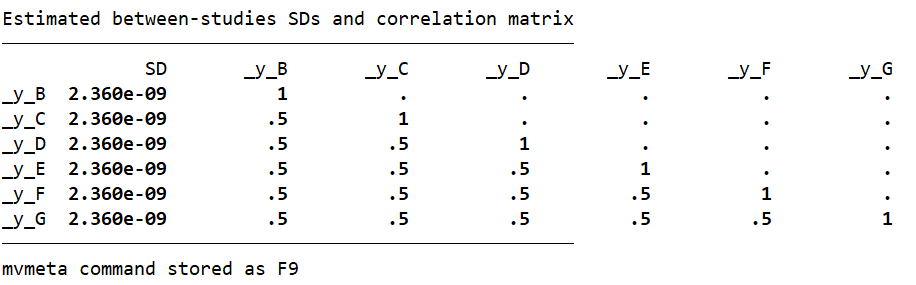


**Supplementary Figure S5.** Consistency assessment of network meta-analysis of FMA - UE - Proximal.

**Design consistency**


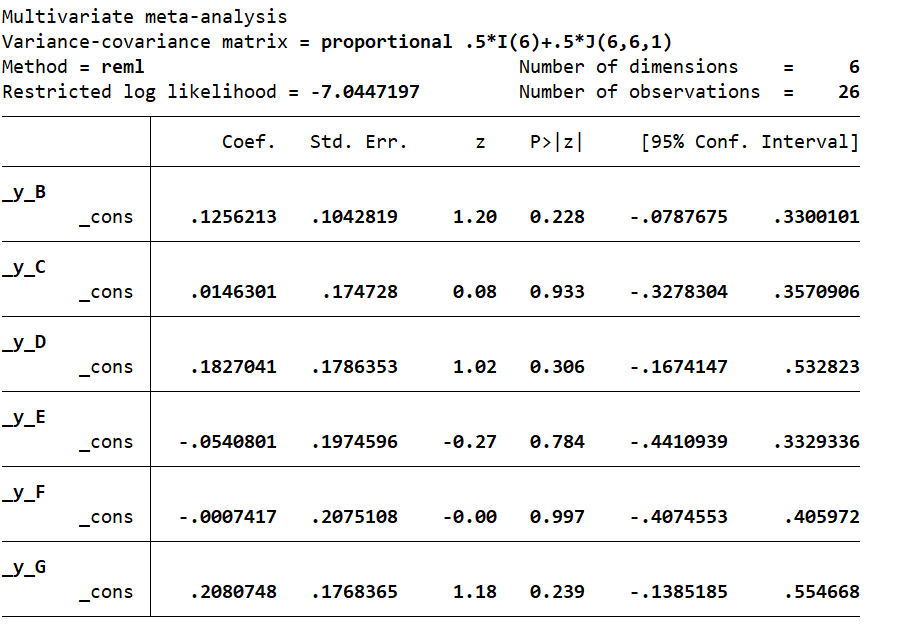


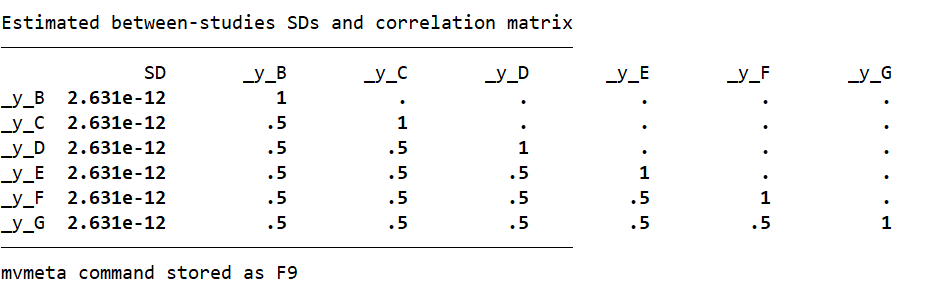


**Supplementary Figure S6.** Consistency assessment of network meta-analysis of FMA - UE - Distal.

**Design consistency**


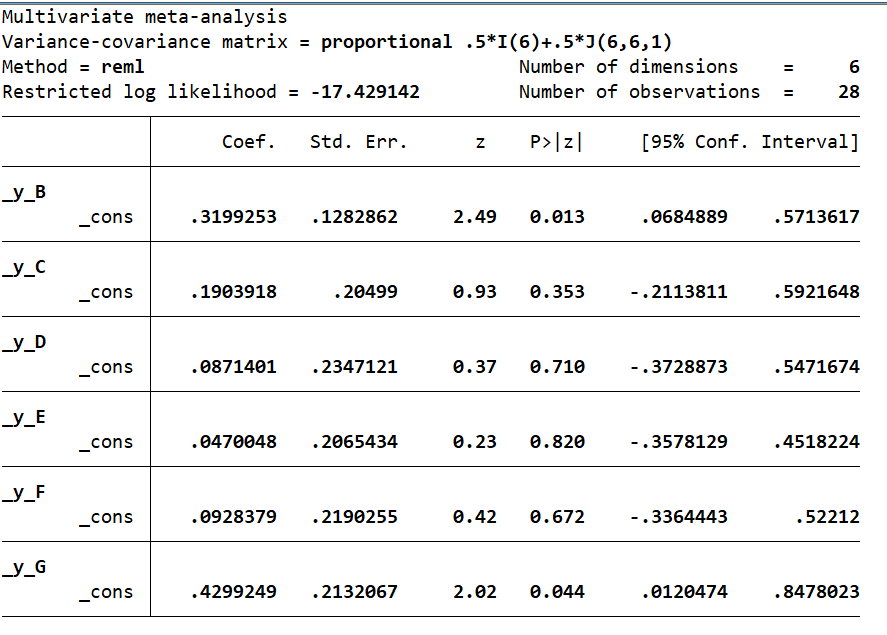


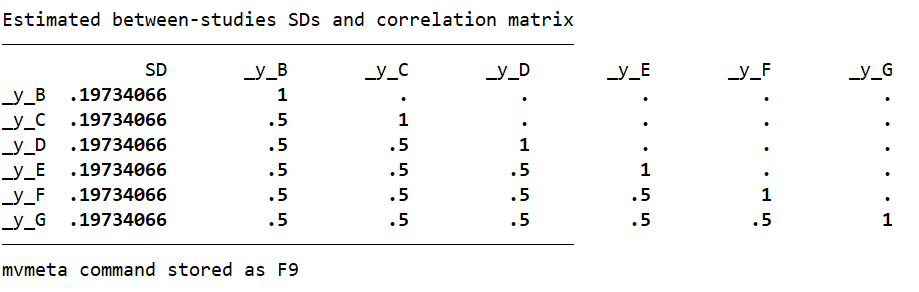


**Supplementary Figure S7.** Consistency assessment of network meta-analysis of ARAT.

**Design consistency**


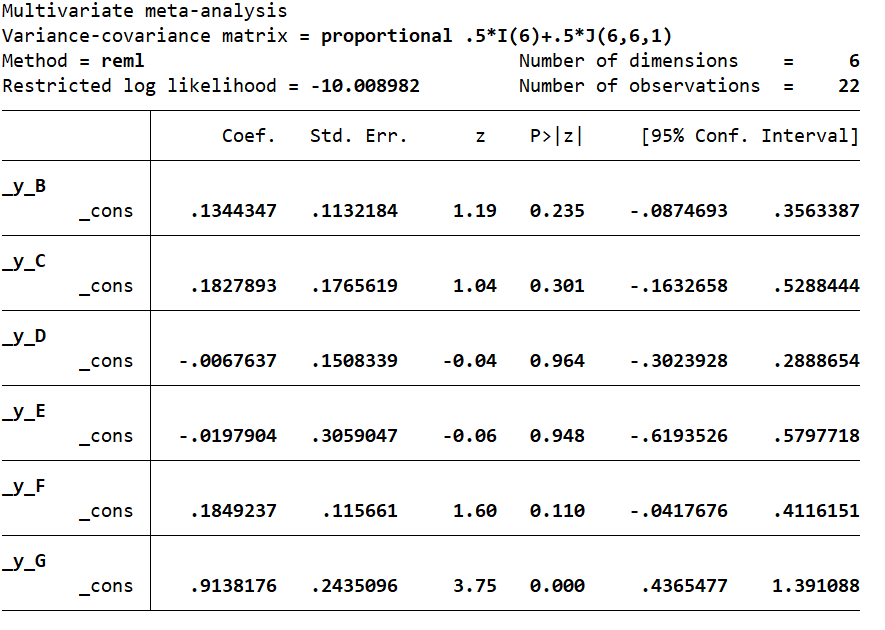


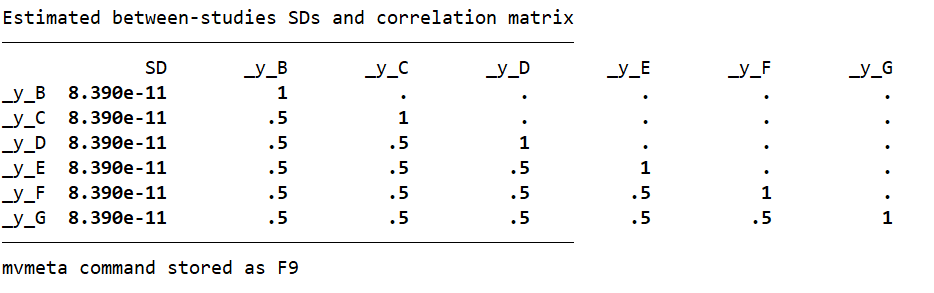


**Supplementary Figure S8:** The Publication Bias and Consistency Assessment in FMA - UE - Proximal.


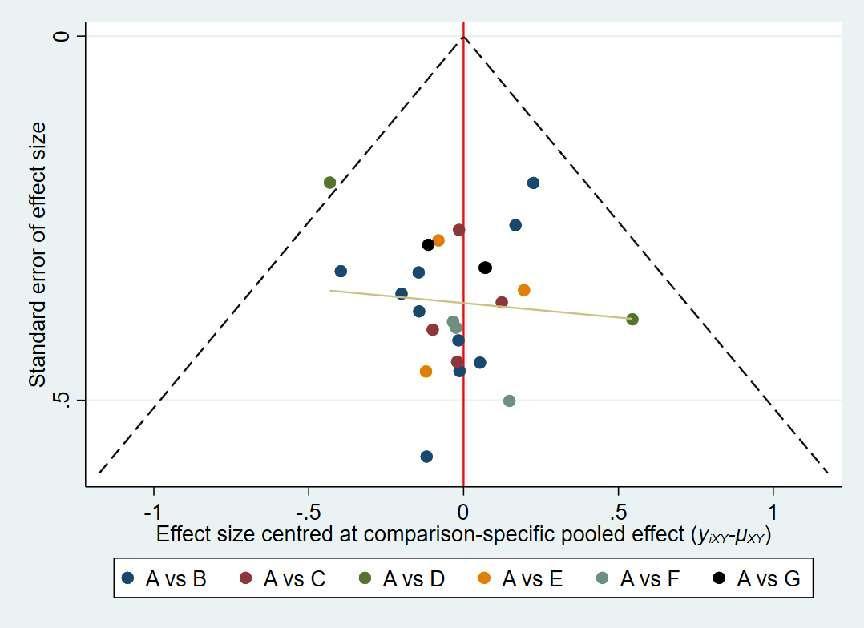


**Supplementary Figure S9**: The Publication Bias and Consistency Assessment in FMA - UE - Distal.


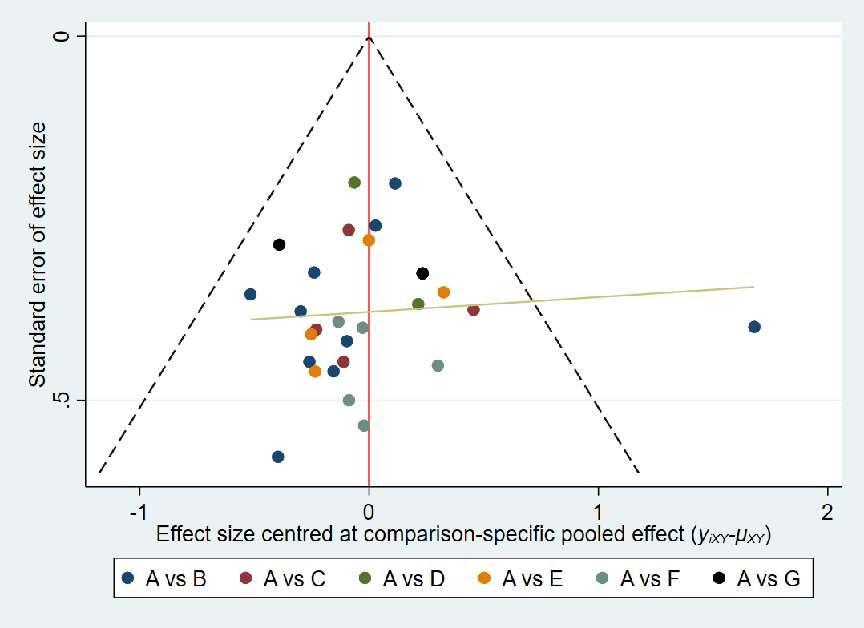


**Supplementary Figure S10:** The Publication Bias and Consistency Assessment in MBI.


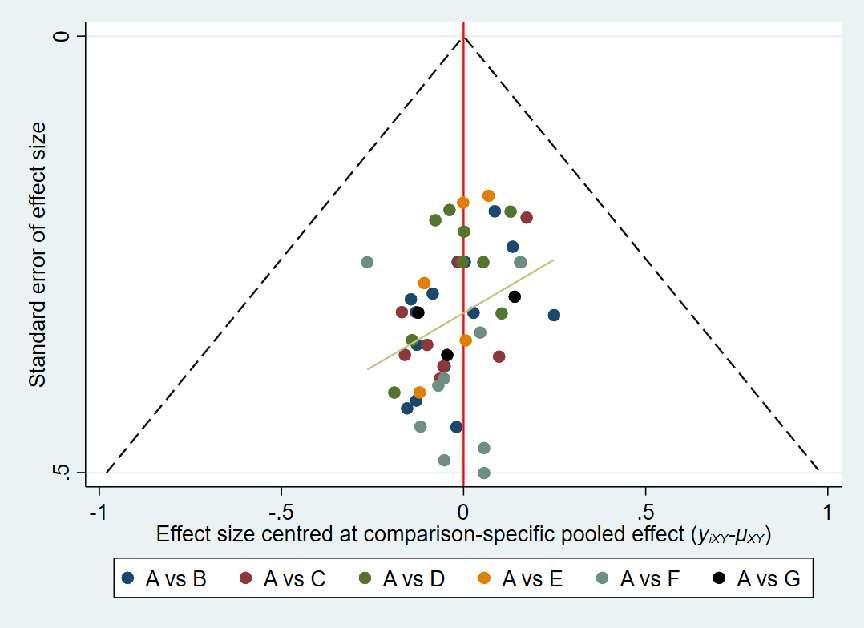


**Supplementary Figure S11 :** The Publication Bias and Consistency Assessment in ARAT.


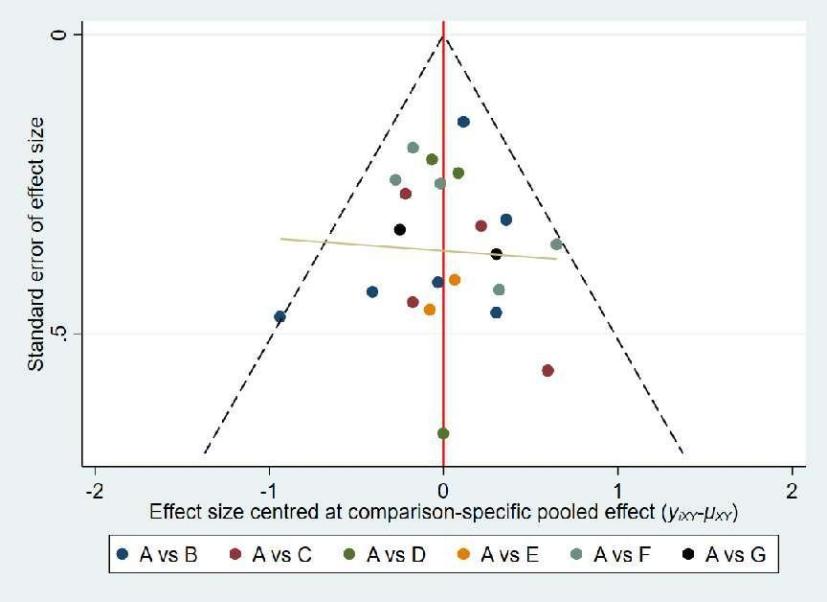

Supplement: Supplementary file 1 [file Data_Sheet_1.docx]
